# *O*-Acetylation of Capsular Polysialic Acid Enables *Escherichia coli* K1 Escaping from Siglec-Mediated Innate Immunity and Lysosomal Degradation of *E. coli*-Containing Vacuoles in Macrophage-Like Cells

**DOI:** 10.1128/spectrum.00399-21

**Published:** 2021-12-08

**Authors:** Jinghua Yang, Wei Ma, Yuanyuan Wu, Hui Zhou, Siyu Song, Yuqi Cao, Chengxu Wang, Xiangyuan Liu, Jinwei Ren, Jinyou Duan, Zhichao Pei, Cheng Jin

**Affiliations:** a State Key Laboratory of Mycology, Institute of Microbiology, Chinese Academy of Sciences, Beijing, China; b University of Chinese Academy of Sciences, Beijing, China; c Shanxi Key Laboratory of Natural Products & Chemical Biology, College of Chemistry & Pharmacy, Northwest A&F University, Yanglin, Shanxi, China; d School of Life Science, Inner Mongolia University, Hohhot, Inner Mongolia, China; e Department of Pharmaceutical Engineering, School of Chemical Engineering and Technology, Tianjin University, Tianjin, China; University of Georgia

**Keywords:** *Escherichia coli* K1, O-acetylation, Siglecs, adherence, invasion, capsular polysialic acid, interaction, intracellular trafficking, macrophage-like cells, pathogenesis

## Abstract

Escherichia coli K1 causes bacteremia and meningitis in human neonates. The K1 capsule, an α2,8-linked polysialic acid (PSA) homopolymer, is its essential virulence factor. PSA is usually partially modified by *O*-acetyl groups. It is known that *O*-acetylation alters the antigenicity of PSA, but its impact on the interactions between E. coli K1 and host cells is unclear. In this study, a phase variant was obtained by passage of E. coli K1 parent strain, which expressed a capsule with 44% *O*-acetylation whereas the capsule of the parent strain has only 3%. The variant strain showed significantly reduced adherence and invasion to macrophage-like cells in comparison to the parent strain. Furthermore, we found that *O*-acetylation of PSA enhanced the modulation of trafficking of E. coli-containing vacuoles (ECV), enabling them to avoid fusing with lysosomes in these cells. Intriguingly, by using quartz crystal microbalance, we demonstrated that the PSA purified from the parent strain interacted with human sialic acid-binding immunoglobulin-like lectins (Siglecs), including Siglec-5, Siglec-7, Siglec-11, and Siglec-14. However, *O*-acetylated PSA from the variant interacted much less and also suppressed the production of Siglec-mediated proinflammatory cytokines. The adherence of the parent strain to human macrophage-like cells was significantly blocked by monoclonal antibodies against Siglec-11 and Siglec-14. Furthermore, the variant strain caused increased bacteremia and higher lethality in neonatal mice compared to the parent strain. These data elucidate that *O*-acetylation of K1 capsule enables E. coli to escape from Siglec-mediated innate immunity and lysosomal degradation; therefore, it is a strategy used by E. coli K1 to regulate its virulence.

**IMPORTANCE** Escherichia coli K1 is a leading cause of neonatal meningitis. The mortality and morbidity of this disease remain significantly high despite antibiotic therapy. One major limitation on advances in prevention and therapy for meningitis is an incomplete understanding of its pathogenesis. E. coli K1 is surrounded by PSA, which is observed to have high-frequency variation of *O*-acetyl modification. Here, we present an in-depth study of the function of *O*-acetylation in PSA at each stage of host-pathogen interaction. We found that a high level of *O*-acetylation significantly interfered with Siglec-mediated bacterial adherence to macrophage-like cells, and blunted the proinflammatory response. Furthermore, the *O*-acetylation of PSA modulated the trafficking of ECVs to prevent them from fusing with lysosomes, enabling them to escape degradation by lysozymes within these cells. Elucidating how subtle modification of the capsule enhances bacterial defenses against host innate immunity will enable the future development of effective drugs or vaccines against infection by E. coli K1.

## INTRODUCTION

Escherichia coli K1 is the most common Gram-negative bacterium which causes neonatal meningitis (1, 2). The important stage of infection occurs upon reaching a high level of bacteremia, which leads to the onset of meningitis (3). Therefore, E. coli must be able to evade the host immune defense system and multiply in the blood. Adherence and invasion, as well as survival in macrophages, thus present important pathogenicity in a bacterial infection. It is well known that the K1 capsule protects E. coli against opsonophagocytosis and complement-mediated killing in the presence of anti-capsule antibody (4–6). Interestingly, E. coli K1 strains have been observed to have a high frequency of variation in acetylated and unacetylated forms of capsule (7). Studies have shown that *O*-acetylation helps bacteria to evade clearance by antibodies against one capsule form, enhances bacterial resistance to desiccation, and hinders the cleavage of sialic acids by neuraminidase (8–11). However, the biological significance of *O*-acetylation in host*-*E. coli K1 interaction is largely unknown.

E. coli K1 polysialic acids (PSAs) are long chains composed of 160 to 230 N-acetylneuraminic acid (Neu5Ac or sialic acid) residues in an α2,8-linkage (12). *O*-acetyl groups randomly decorate the carbon-7 or carbon -9 hydroxyl on the exocyclic chain of Neu5Ac at a high frequency of 1:50 to 1:20, and can migrate non-enzymatically from carbon-7 to carbon -9 (7). *O*-acetyl modification increases capsule surface hydrophobicity and affects the capsule’s physiochemical properties, which may create new ligands or mask existing ligands (13, 14). It is known that *O*-acetylation regulates the function of the terminal sialic acid of glycoconjugate ligands in mammalian cells (15). Therefore, evaluating the potential influence of *O*-acetylation of PSA upon sialic acid-mediated host-pathogen interaction is important for understanding E. coli K1 infection.

The sialic acid-containing ligands can be recognized by sialic acid-binding immunoglobulin-like lectins (Siglecs), a superfamily of lectins expressed on leukocytes, which regulate the innate immunity and inflammatory activities of cells (16, 17). Siglecs are composed of extracellular immunoglobulin domains, including an amino-terminal V-set domain that contains the sialic acid-binding site (18). The cytoplasmic domains of most Siglecs have immunoreceptor tyrosine-based inhibitory motif (ITIM)-generating inhibitory signaling via the recruitment of tyrosine phosphatases SHP-1 and SHP-2 to attenuate immune response (17, 19, 20). A few Siglecs (i.e., Siglec-14/15/16) which evolved in humans associate with the immunoreceptor tyrosine-based activation motif (ITAM)-coupled adaptor, DAP12, to generate activating signal through the recruitment of spleen tyrosine kinase, and promote a proinflammatory response (17, 21, 22). In order to escape immune attack, some bacteria have evolved molecular mimicry of host self-associated molecular patterns, which are recognized by inhibitory Siglecs (18, 23–26). For instance, E. coli K1 PSA binds to inhibitory Siglec-11 on microglia in the human brain by molecular mimicry of the neural cell adhesion molecule (19). This in-*trans* interaction via Siglec-11 reduces the inflammatory neurotoxicity of phagocytes and blocks the reactivity of mononuclear phagocytes (19, 20, 27). As a response to this influence, the activation of Siglec-16 generates protective inflammatory responses and increases the elimination of this pathogen (28). The paired Siglecs, such as Siglec-11/16 and Siglec-5/14, display nearly identical extracellular ligand-binding domains, but have intracellular motifs with opposing signaling functions (21, 28). The balanced activities of the paired Siglecs provide maximal protection from bacterial infection with minimal immunopathology (22).

In E. coli K1, the *O*-acetylation of PSA is regulated by two acetyl-CoA-dependent acetyltransferases, NeuD and NeuO (29, 30). NeuD acetylates monomeric sialic acids and accounts for 2 to 4% of natural *O*-acetylation of the K1 capsule (31). Interestingly, E. coli K1 strains harboring the prophage CUS-3 express the *neuO* gene in a unique contingency locus (32). NeuO preferentially modifies polysialic acid chains and accounts for the majority of *O*-acetylation (33–35). The *neuO* gene mediates the random translational switch by the slip-strand DNA mispairing of tandem repeats (5′-AAGACTC-3′)_n_ at the locus (32, 34). Only repeat numbers (“n”) that are multiples of three allow for full-length translation of *neuO*, and the strain displays phase “on.” In all other instances, the translational frame shifts and the strain is switched to phase “off.” The catalytic efficiency or stability of NeuO is controlled by extension in the length of poly-ψ domains (33). Therefore, the variation of tandem repeat numbers not only leads to a reversible phase switch but also generates a large number of phase variants with different levels of *O*-acetylation (32, 35).

In this study, we obtained a phase “on” variant strain which contained 44% *O*-acetylated PSA. We demonstrate that elevated *O*-acetylation attenuates bacterial binding to macrophage-like cells by interfering with the recognition between PSAs and specific Siglec receptors. *O*-acetylated PSA also significantly suppresses the Siglec-mediated secretion of proinflammatory cytokines. Furthermore, the *O*-acetylation of PSA modulates the trafficking of *E. coli*-containing vacuoles (ECVs), allowing them to escape killing by lysosomes within these cells. These findings reveal the molecular mechanism by which *O*-acetylation of PSA regulates the interaction of E. coli K1 with macrophage-like cells, enhancing bacterial pathogenicity.

## RESULTS

### Generation of the variant strain and the capsule-deleted mutant strain.

**FIG 1:**
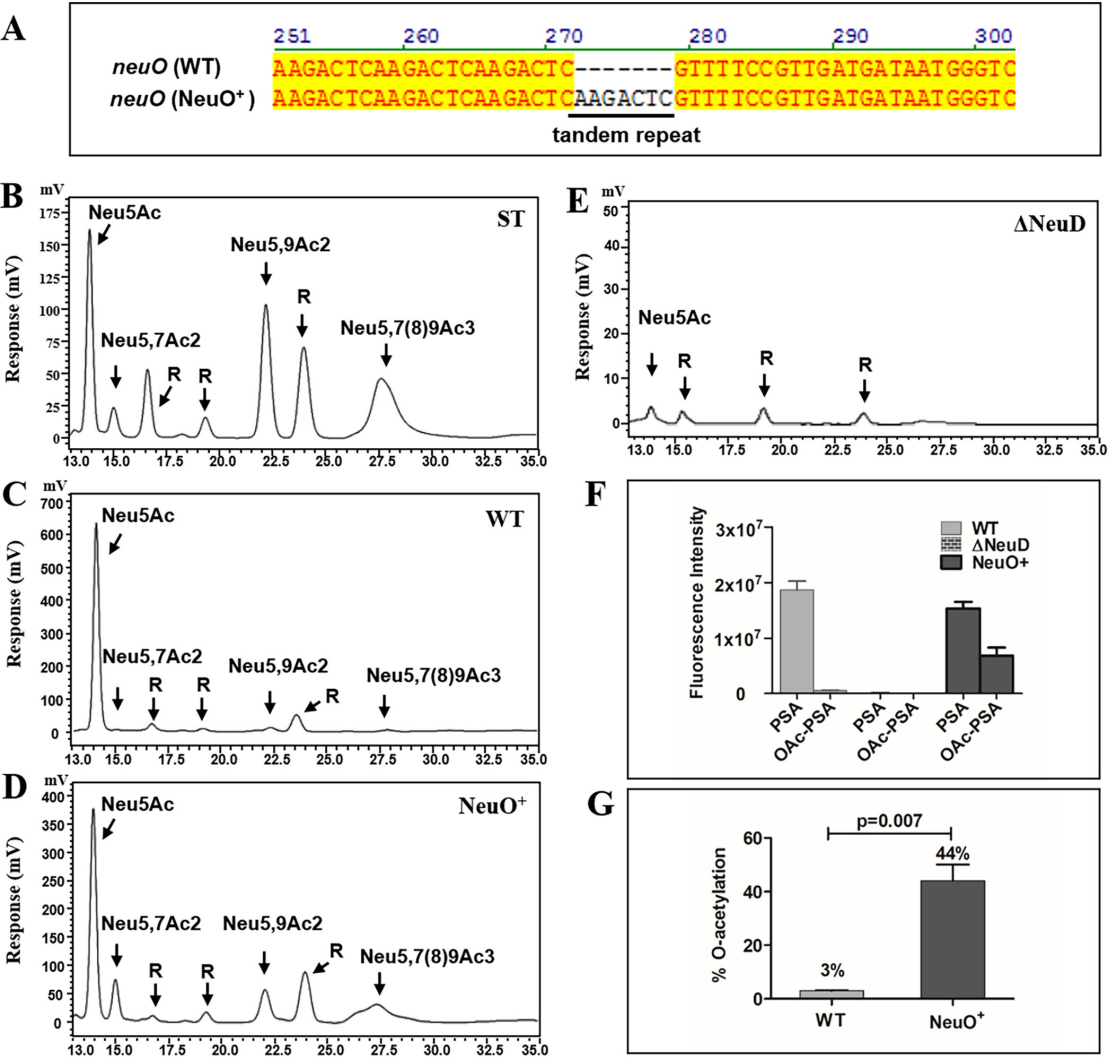
Genetic variation of *neuO* and DMB-HPLC analysis of capsular polysialic acids. (A) Comparison of 5′-end of the *neuO* genes of the WT and variant. The *neuO* of the WT contained 38 tandem repeats and was inactive because of the slip-strand DNA mutation. By gain of one tandem repeat, the *neuO* gene in the variant was fully translated into active acetyltransferase. (B) HPLC analysis of sialic acids standards (ST). Neu5Ac represents *N*-acetylneuraminic acid. Neu5,7Ac2 represents carbon position 7 *O*-acetylated Neu5Ac. Neu5,9Ac2 represents carbon position 9 *O*-acetylated Neu5Ac. Neu5,7(8)9Ac_3_ represents carbon position 7, 8, and 9 *O*-acetylated Neu5Ac. R represents the reagent peak of unknown identity. (C-E) HPLC analysis of extracellular PSAs isolated from the WT, NeuO^+^ variant, and Δ*neuD* mutant. The peaks were assigned referring to the corresponding standards. (F) Total amounts of PSAs and *O*-acetylated PSAs (OAc-PSA) from the WT, Δ*neuD*, and NeuO^+^. (G) The level of *O*-acetylation of PSAs from the WT and NeuO^+^ strains. Data are shown as mean ± SD, and were analyzed by a two-tailed paired Student's *t* test using Prism software. Error bars indicate median for values from three or four separate experiments.

Because the *O*-acetylation of E. coli K1 PSA is mainly governed by *neuO* carried on prophage CUS-3 (35), we searched through sequences in GenBank and found that some of the *neuO* genes were located between the endosialidase gene *sialK1* and the integrase gene *int*. The primers (see Table S1 in the supplemental material) were designed based on these two genes, and the *neuO* gene of the parent strain (WT) was amplified and sequenced. The result showed that the *neuO* of the WT contained 38 tandem repeats and was inactive; therefore, the WT displays phase “off.” Previous studies have shown that 1 to 2% of E. coli K1 strains changed phase during growth in LB medium over four generations (36). Thus, to generate a phase “on” variant, the WT was propagated in LB and passaged four times. After screening 30 single colonies through sequencing of *neuO* genes, two colonies were found to contain 39 copies of tandem repeats at the 5′-coding region of *neuO*. Obviously, the WT which acquired one heptanucleotide tandem repeat (5′-AAGACTC-3′) and was turned into the phase “on” variant was designated the NeuO^+^ strain (Fig. 1A). In addition, to generate the capsule-negative mutant, the *neuD* gene was deleted by the one-step method and verified by sequencing, as described in Materials and Methods.

### Quantification of *O*-acetylation of PSAs.

To quantify the K1 capsule and the level of *O*-acetylation, the PSAs were isolated from the WT and the NeuO^+^ strains, respectively. The released monomeric sialic acids were derivatized with 1,2-diamino-4,5-methylenedioxybenzene (DMB) and analyzed using reverse high-pressure liquid chromatography (HPLC). Referring to these standards (Fig. 1B), the PSA of the WT contained mainly Neu5Ac residues and a small amount of *O*-acetylated Neu5Ac residues (Fig. 1C). In comparison with the WT, higher levels of Neu5,7Ac_2_, Neu5,9Ac_2_, and Neu5,7(8)9Ac_3_ were detected in the PSA of the NeuO^+^ variant (Fig. 1D). The trace amount of Neu5Ac residues in the Δ*neuD* mutant suggested the loss of K1 capsule (Fig. 1E). By calculating the peak areas, the relative amounts of PSAs were determined to be 1.8 × 10^7^ and 1.5 × 10^7^, while the relative amounts of *O*-acetylated PSAs were 5.3 × 10^5^ and 6.8 × 10^6^ in the WT and NeuO^+^, respectively (Fig. 1F). Thus, the WT strain contained 3% *O*-acetylation, which was consistent with previous reports (29), and the NeuO^+^ strain contained 44% *O*-acetylation (Fig. 1G), which was almost equally distributed at the C7 and C9 positions and to some extent at the C8 position (Fig. 1D). The amount of PSAs was reduced a little in the variant (Fig. 1F). On the other hand, the relative amount of intracellular sialic acids was determined to be 1.0 × 10^6^ in the WT (see Fig. S1A and 1C) and 2.3 × 10^6^ in the NeuO^+^ (see Fig. S1B and C). Therefore, the total amount of sialic acids was almost identical across the two strains. The intracellular sialic acids were highly *O*-acetylated (see Fig. S1D), suggesting that *O*-acetylation might affect PSAs transported to the outside of the membrane.

We further identified the *O*-acetylation of the two PSAs isolated from the WT and the NeuO^+^ strains by nuclear magnetic resonance (NMR) analysis (see Fig. S2). The one-dimensional ^1^H NMR spectra showed that peaks appeared from 1.5 ppm to 4.3 ppm. The proton areas corresponded to the ring protons and methyl protons of the acetyl groups and to H-3 of Neu5Ac (see Fig. S2A). The peaks between 2.07 ppm and 2.09 ppm were assigned *N*-acetyl signals. The peaks between 2.13 ppm and 2.20 ppm, and between 1.98 ppm and 2.03 ppm, were assigned *O*-acetyl signals, respectively (see Fig. S2B). The two NMR spectra were comparable, although *O*-acetyl groups changed the chemical shifts of ^1^H resonances at the point of substitution. The amount of *O*-acetyl groups in the NeuO^+^ PSA was about 10 times that of the WT PSA, as determined by comparison with the internal standard compound. However, the level of *O*-acetylation of polysaccharide could not be accurately qualified by ^1^H NMR analysis.

### *O*-acetylation of K1 capsule attenuated bacterial adherence and invasion to macrophage-like cells.

**FIG 2:**
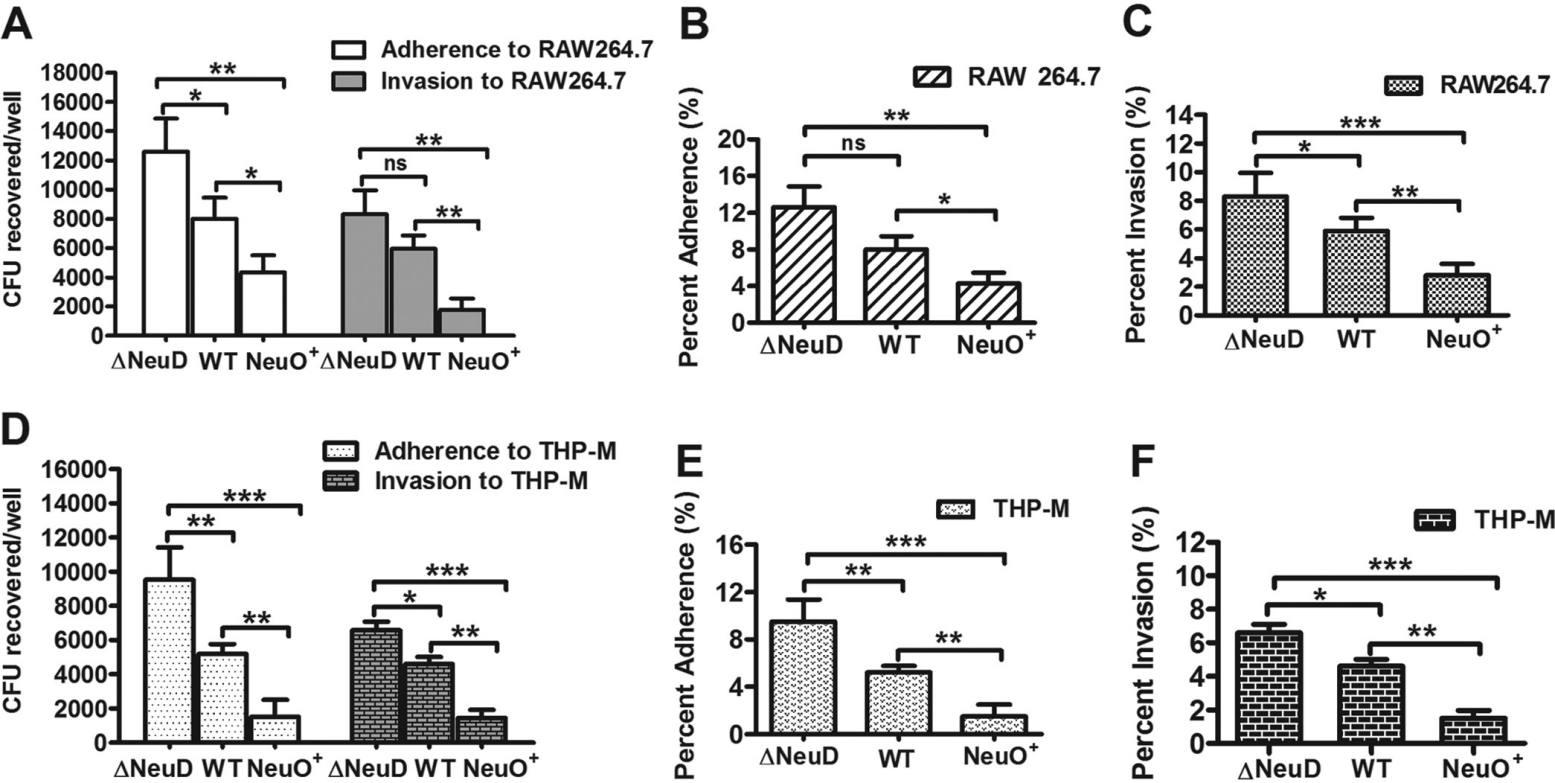
Adherence and invasion of E. coli strains to macrophage-like cells. (A) The total cell-associated bacteria and the internalized bacteria to RAW 264.7. Monolayers (10^5^ cells) were incubated with the WT, Δ*neuD*, and NeuO^+^ (MOI of 1), respectively, and the adherence and invasion of each strain to cells was analyzed. (B) The binding rates of the WT, Δ*neuD*, and NeuO^+^ to RAW 264.7 were compared. (C) The invasion rates of the WT, Δ*neuD*, and NeuO^+^ to RAW 264.7 were compared. (D) The total cell-associated bacteria and the internalized bacteria to THP-M cells. Monolayers (10^5^ cells) were incubated with the WT, Δ*neuD*, and NeuO^+^ (MOI of 10). (E) The binding rates of the WT, Δ*neuD*, and NeuO^+^ to THP-M cells were compared. (F) The invasion rates of the WT, Δ*neuD*, and NeuO^+^ to THP-M cells were compared. Data are shown as mean ± SD, and analyzed by a two-tailed unpaired Student's *t* test (*n* = 4 or 5). ns, *P* > 0.05; *, *P* < 0.05; **, *P* < 0.005; ***, *P* < 0.0005.

In order to reveal the function of PSA *O*-acetylation in host-pathogen interaction, we investigated the adherence and invasion of E. coli K1 strains to the murine macrophage-like cell, RAW 264.7, and the human macrophage-like cell, THP-M, that differentiated from THP-1 monocytes by stimulation with porbol myristate acetate. We demonstrated that the numbers of bacteria binding to RAW 264.7 were 12,600 CFU/well for the Δ*neuD*, 8,017 CFU/well for the WT, and 4,317 CFU/well for the NeuO^+^ (Fig. 2A). The numbers of bacteria internalized into RAW 264.7 were 8,300 CFU/well for the Δ*neuD*, 5,940 CFU/well for the WT, and 2,760 CFU/well for the NeuO^+^ (Fig. 2A). Accordingly, the adherence ratio was 12.6% for the Δ*neuD*, 8.0% for the WT, and 4.3% for the NeuO^+^ (Fig. 2B), while the invasion ratio was 8.3% for the Δ*neuD*, 5.9% for the WT, and 2.8% for the NeuO^+^ (Fig. 2C). When THP-M cells were used, the number of bacteria binding to the cells was 9,538 CFU/well for the Δ*neuD*, 5,205 CFU/well for the WT, and 1,518 CFU/well for the NeuO^+^ (Fig. 2D). The numbers of internalized bacteria were 6,567 CFU/well for the Δ*neuD*, 4,600 CFU/well for the WT, and 1,463 CFU/well for the NeuO^+^ (Fig. 2D). Therefore, the adherence ratio was 9.5% for the Δ*neuD*, 5.2% for the WT, and 1.5% for the NeuO^+^ (Fig. 2E), whereas the invasion ratio was 6.6% for the Δ*neuD*, 4.6% for the WT, and 1.5% for the NeuO^+^ (Fig. 2F). These data demonstrate that the capsule-negative mutant adhered to macrophage-like cells in greater numbers than the encapsulated strains, but the increase level was less than 2-fold, which was consistent with the binding of PSA-negative mutants of Neisseria meningitides to buccal epithelial cells (37). Interestingly, the adherence of the NeuO^+^ to macrophage-like cells was reduced by 2 to 3 times compared to that of the WT, indicating that *O*-acetylation of the K1 capsule can effectively interfere with bacterial binding, especially in human THP-M cells.

### *O*-acetylated K1 capsule enhanced the modulation of ECV trafficking to avoid fusion with lysosomes in macrophage-like cells.

**FIG 3:**
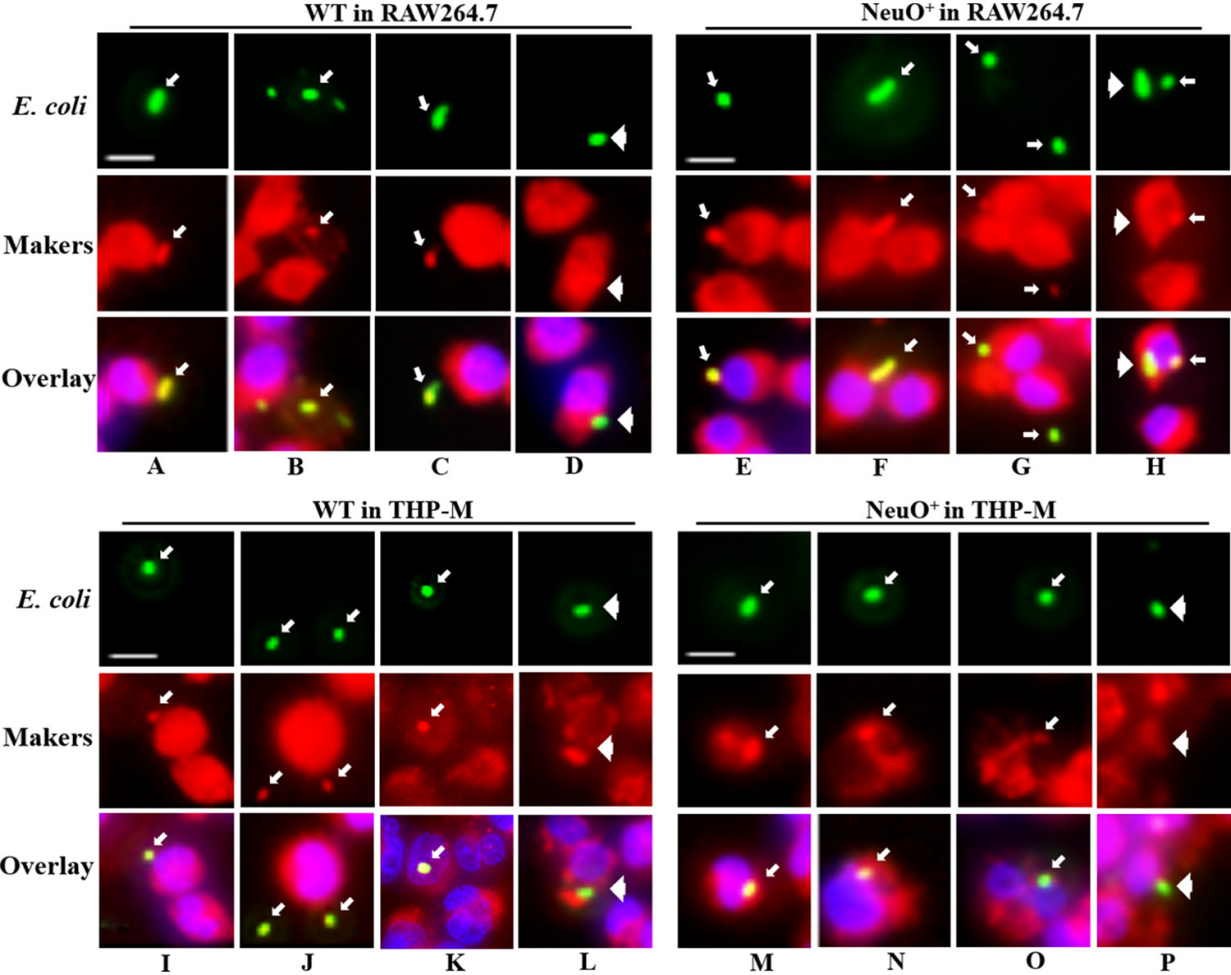
Intracellular trafficking of E. coli*-*containing vacuoles (ECVs) in macrophage-like cells. RAW 264.7 cells and THP-M cells were grown on chamber slides and incubated with FITC-labeled WT strain or NeuO^+^ strain, respectively. The monolayers were immunostained with antibodies against the early endosomal marker, EEA1 (A, E, I, M), at 30 min incubation; antibodies against either the late endosomal marker Rab7 (B, F, J, N) or the pre-lysosomal marker Lamp-1 (C, G, K, O) at 60 min incubation; and antibodies against the lysosomal marker, cathepsin D (D, H, L, P), at 90 min incubation. The presence of primary antibodies was revealed using Alexa-594-conjugated secondary antibody. Presence of each endocytic markers on ECVs was determined by overlay of images obtained from two channels. All markers were recruited on ECVs (arrows), but cathepsin D was not present on some of ECVs (arrowheads), indicating that these ECVs were not delivered to lysosomes and thus escaped elimination. Scale bars, 10 μm.

Previous study showed that K1 capsule modulated the trafficking of E. coli-containing vacuoles (ECVs) and enhanced intracellular bacterial survival in human brain microvascular endothelial cells (38). Given that the survival of E. coli K1 within phagocytic cells plays an important role in the development of bacteremia, which is crucial for E. coli crossing of the blood-brain barrier (39), we investigated the effect of capsular *O*-acetyl modification on the trafficking of ECVs inside macrophages. To this end, fluorescein isothiocyanate (FITC)-labeled E. coli was incubated with RAW 264.7 or THP-M cells, and the fusion of ECVs with endosomes or lysosomes was revealed by the presence of antibodies against specific markers and Alexa Fluor 594-conjugated antibody. Double-immunofluorescence staining revealed that early-endosomal auto-antigen 1 (EEA1) at 30 min incubation (Fig. 3A, E, I and M), and late endosomal compartments Rab7 GTPase (Fig. 3B, F, J and N), as well as prelysosomal-associated protein (Lamp-1) at 60 min of incubation (Fig. 3C, G, K and O) were present in all of the WT- and NeuO^+^-containing ECVs, indicating that all of the ECVs were fused with early and late endosomes as well as prelysosomes. However, after 90 min of incubation with RAW 264.7 cells (Fig. 3D and H), 30% of the WT-containing ECVs (6 out of 20 ECVs observed) and 40% of the NeuO^+^-containing ECVs (8 out of 20 ECVs observed) did not acquire cathepsin D, a hydrolytic enzyme in lysosome. After 90 min of incubation with THP-M cells (Fig. 3L and P), 50% of the WT-containing ECVs (10 out of 20 ECVs observed) and 85% of the NeuO^+^-containing ECVs (17 out of 20 ECVs observed) did not acquire cathepsin D. These results suggested that these ECVs were arrested at the stage prior to fusion with lysosomes and thus escaped killing by lysozymes.

**FIG 4:**
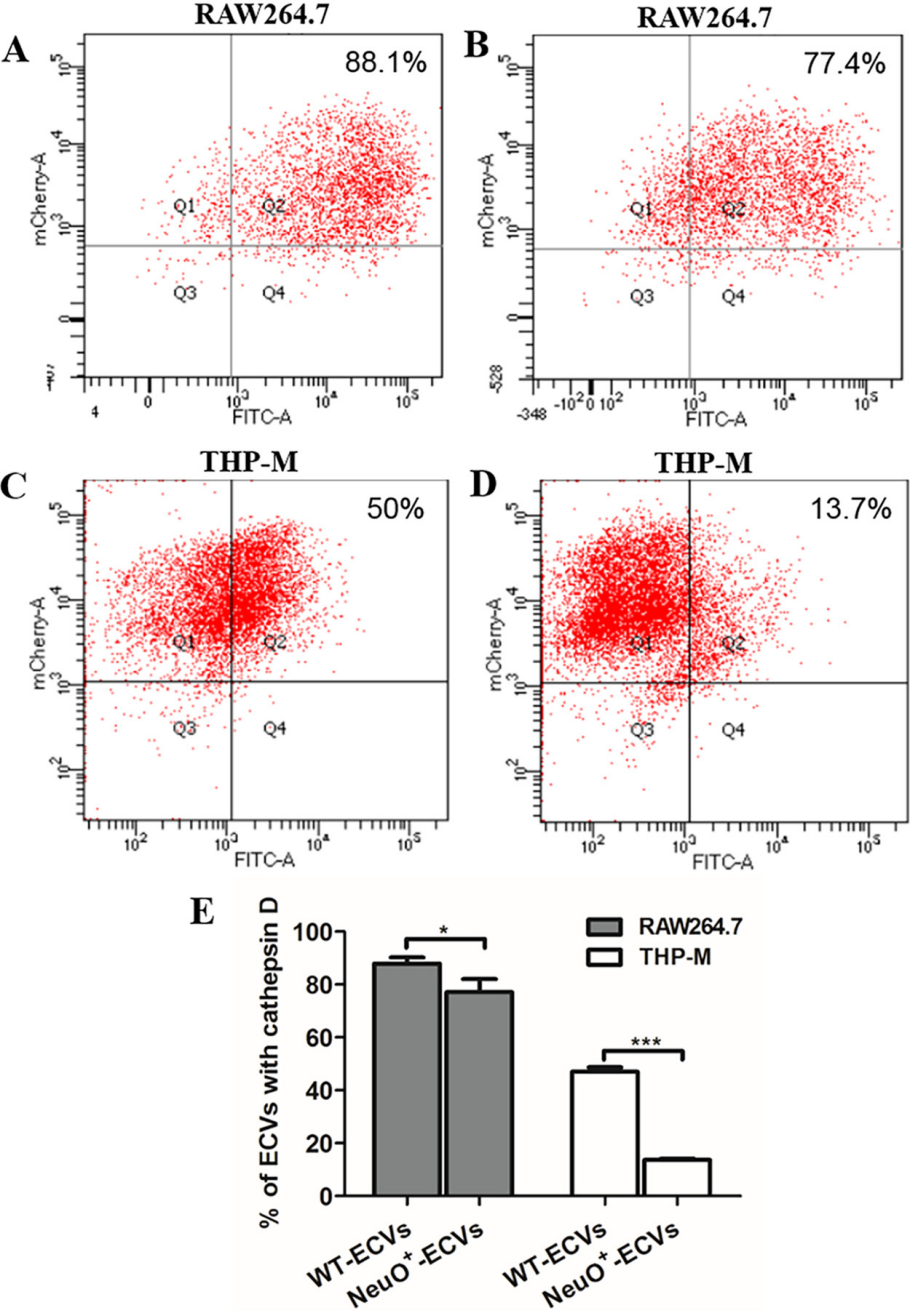
Epitope-specific flow cytometry analysis of ECVs in macrophage-like cells. RAW 264.7 cells were incubated with FITC-labeled WT (A) or NeuO^+^ (B). THP-M cells were incubated with FITC-labeled WT (C) or NeuO^+^ (D). The postnuclear supernatant (PNS) was prepared and immunostained with antibodies recognizing the cytoplasmic domains of cathepsin D, a lysozyme marker. The presence of primary antibodies was revealed with Alexa 594-conjugated secondary antibodies. Data were acquired by flow cytometry, and the gating thresholds of green line and red line were set as described in Materials and Methods. The percentage of ECVs which were positive for cathepsin D was plotted as indicated in graphs (E). Each experiment was carried out at three separate times. Data are shown as mean ± SD and were analyzed by a two-tailed unpaired Student's *t* test. *, *P* < 0.05; ***, *P* < 0.0001.

To precisely detect cathepsin D markers on the ECVs, we performed an epitope-specific flow cytometry assay. The postnuclear supernatants (PNS) were prepared from either RAW 264.7 cells or from THP-M cells infected with FITC-labeled E. coli K1 strains and incubated with antibodies against cathepsin D prior to flow cytometry analysis. The results revealed that 88.1% of the WT-containing ECVs and 77.4% of the NeuO^+^-containing ECVs acquired cathepsin D on RAW 264.7 cells (Fig. 4A and B), while 50% of the WT-containing ECVs and 13.7% of the NeuO^+^-containing ECVs acquired cathepsin D on THP-M cells (Fig. 4C and D). Thus, the numbers of NeuO^+^-containing ECVs delivered to lysosomes were fewer than the numbers of WT-containing ECVs (Fig. 4E), indicating that more of the NeuO^+^ strains escaped killing by lysozymes in lysosomes, especially in human macrophage-like cells. These findings corroborated the results of immunofluorescence observations and the analysis of counting fused ECVs (Fig. 3). Taken together, these results show that *O*-acetyl modification of the K1 capsule modulated trafficking of ECVs to avoid fusion with lysosomes, which might enhance intracellular bacterial survival in macrophage-like cells.

### *O-*acetylation of K1 capsule attenuates *E. coli* K1 binding to Siglecs.

**FIG 5:**
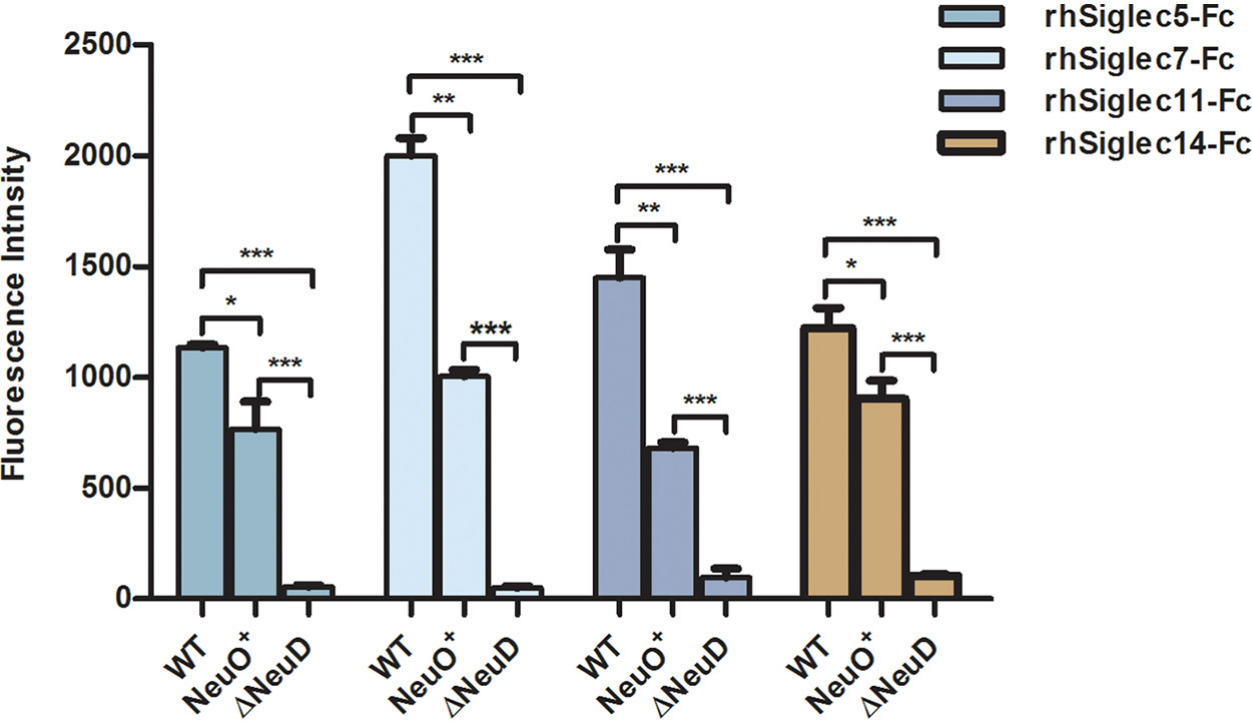
The binding of E. coli K1 strains to Siglecs. The human CD33 rSiglec-Fc chimeras (i.e., rhSiglec-5/7/11/14-Fc) were separately plated on a protein A-coated well and incubated with FITC-labeled E. coli strains WT, Δ*neuD*, and NeuO^+^, respectively. Bindings between bacteria and Siglec were examined and quantified by fluorescence intensity. Each experiment was carried out at three separate times, in triplicate. Data are shown as mean ± SD and were analyzed by a two-tailed unpaired Student's *t* test. *, *P* < 0.05; **, *P* < 0.005; ***, *P* < 0.0005.

To understand how *O*-acetylation of K1 capsule affects E. coli adherence to host cells, we investigated the binding between E. coli K1 strains and Siglecs which specifically recognize sialic acid-containing ligands. In humans, 15 Siglecs have been defined as expressing on different immune cells, and each Siglec prefers to recognize a unique sialic acid-containing ligand (17, 18). Only a few Siglecs, including Siglec-5/14, Siglec-11/16, and Siglec-7 are supposed to recognize the terminal α2,8-linked di-sialic acids of oligosaccharides (17, 18, 22, 40). The K1 capsule is a α2,8-linked polysialic acid long chain, therefore, we investigated the binding of K1 capsule to these few Siglecs. In initial studies, we evaluated the binding of FITC-labeled E. coli K1 strains to the recombinant human Siglec-Fc chimeras (rhSiglec-Fc) by fluorescence intensity. We found that the K1 capsule-positive strains were able to recognize Siglec-5, Siglec-7, Siglec-11, and Siglec-14 at different levels, while the capsule-negative mutant Δ*neuD* lost ability to bind to these Siglecs (Fig. 5). This result suggested that K1 capsular polysialic acid might be the ligand recognized by these Siglec receptors. Interestingly, the binding of the highly *O*-acetylated NeuO^+^ strain to each of these Siglecs was significantly reduced compared with that of the WT strain. This result suggested that *O*-acetylation of the K1 capsule diminished the binding of E. coli K1 to the Siglecs that specifically recognized α2,8-linked sialic acids.

### *O*-acetylation of K1 capsule interferes with the interaction between PSA and specific Siglecs.

**FIG 6:**
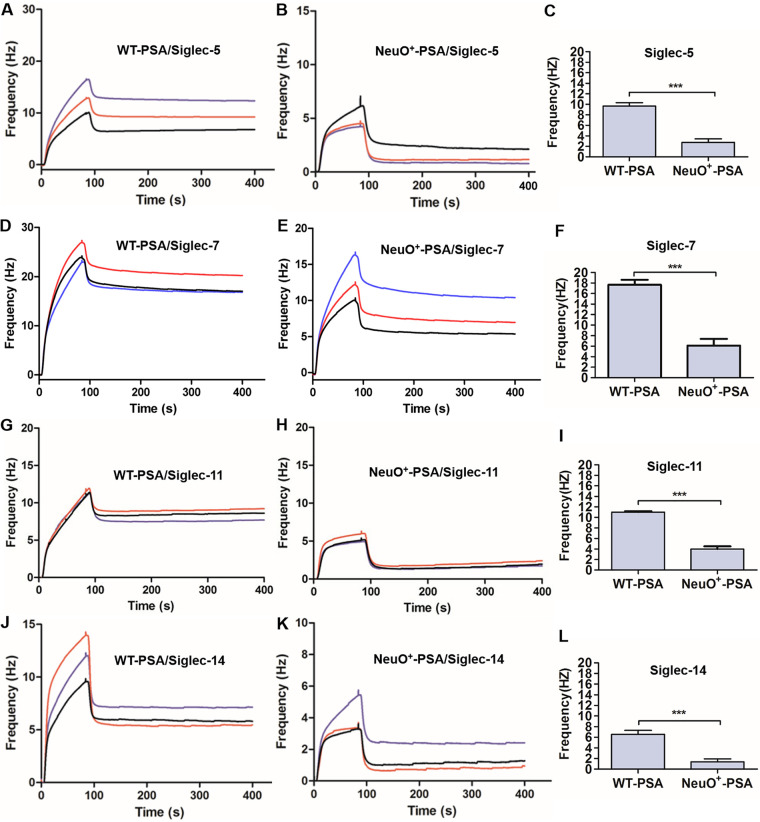
The direct interaction between purified PSAs and Siglecs as detected by a Quartz Crystal Microbalance (QCM) Biosensor. (A) The interaction between WT PSA and rhSiglec-5-Fc. (B) The interaction between NeuO^+^ PSA and rhSiglec-5-Fc. (C) The frequency responses of the two PSAs to rhSiglec-5-Fc were compared. (D) The interaction between WT PSA and rhSiglec-7-Fc. (E) The interaction between NeuO^+^ PSA and rhSiglec-7-Fc. (F) The frequency responses of the two PSAs to rhSiglec-7-Fc were compared. (G) The interaction between WT PSA and rhSiglec-11-Fc. (H) The interaction between NeuO^+^ PSA and rhSiglec-11-Fc. (I) The frequency responses of the two PSAs to rhSiglec-11-Fc were compared. (J) The interaction between WT PSA and rhSiglec-14-Fc. (K) The interaction between NeuO^+^ PSA and rhSiglec-14-Fc. (L) The frequency responses of the two PSAs to rhSiglec-14-Fc were compared. Purified capsular polysaccharides were immobilized on the surface of chips, and each rSiglec-Fc was injected three times over the chip surface. The binding was evaluated by QCM. Curves in colors indicate three independent experiments performed. Data were analyzed by a two-tailed unpaired Student's *t* test (*n* = 3). ***, *P* < 0.0001.

Further testing was performed to determine the direct interaction between purified polysialic acids and specific Siglecs using quartz crystal microbalance (QCM). First, the polysialic acids were isolated and purified from the WT and the NeuO^+^, respectively. The two PSAs exhibited smearing bands (see Fig. S3A), suggesting high molecular weight. A linearity between the retention time and the logarithmic values of molecular weights of dextran standards was acquired using high-performance gel permeation chromatography (HPGPC) (see Fig. S3B). The single peak detected in each sample indicated that the PSAs were pure. The retention time for WT PSA and NeuO^+^ PSA was 38.451 min and 38.684 min (see Fig. S3C and D), respectively, and their average molecular weights were calculated as 74.9 kDa and 67.8 kDa, respectively, referring to the standards. Thus, the maximum chain lengths of the two PSAs were estimated to be more than 200 sialic acids. Second, to verify that the purified PSAs were free of lipid-A, they were examined by ^31^P NMR analysis, because lipid-A contains glycosidic diphosphate moiety. The NMR data (see Fig. S4) clearly showed that ^31^P signals were not present in the spectra of the purified PSAs, while two phosphate signals were present in the spectrum of lipopolysaccharide (LPS) containing lipid-A. Therefore, the PSAs purified from the WT and NeuO^+^ strains were free of lipid-A. Finally, an equal amount of the purified PSAs (10 μM) was injected on the chip immobilized with Siglecs, and frequency shifts were observed from the interactions between PSA and each of the Siglecs. The results directly demonstrated that PSAs purified from the WT and the NeuO^+^ interacted with Siglec-5 (Fig. 6A and B), Siglec-7 (Fig. 6C and D), Siglec-11 (Fig. 6G and H), and Siglec-14 (Fig. 6J and K). Importantly, the frequency responses from the interaction between highly *O*-acetylated PSA and Siglecs were significantly reduced (by 3 to 4 times) compared to the frequency responses from the interaction between PSA and Siglecs (Fig. 6C, F and I and L). Taken together, *O*-acetylation of K1 capsule interfered with the interaction between α2,8-linked PSA ligands and their specific Siglec receptors, and thus attenuated the adherence of E. coli K1 to immune cells.

### *O*-acetylated PSA suppressed the expression and secretion of proinflammatory cytokines.

**FIG 7:**
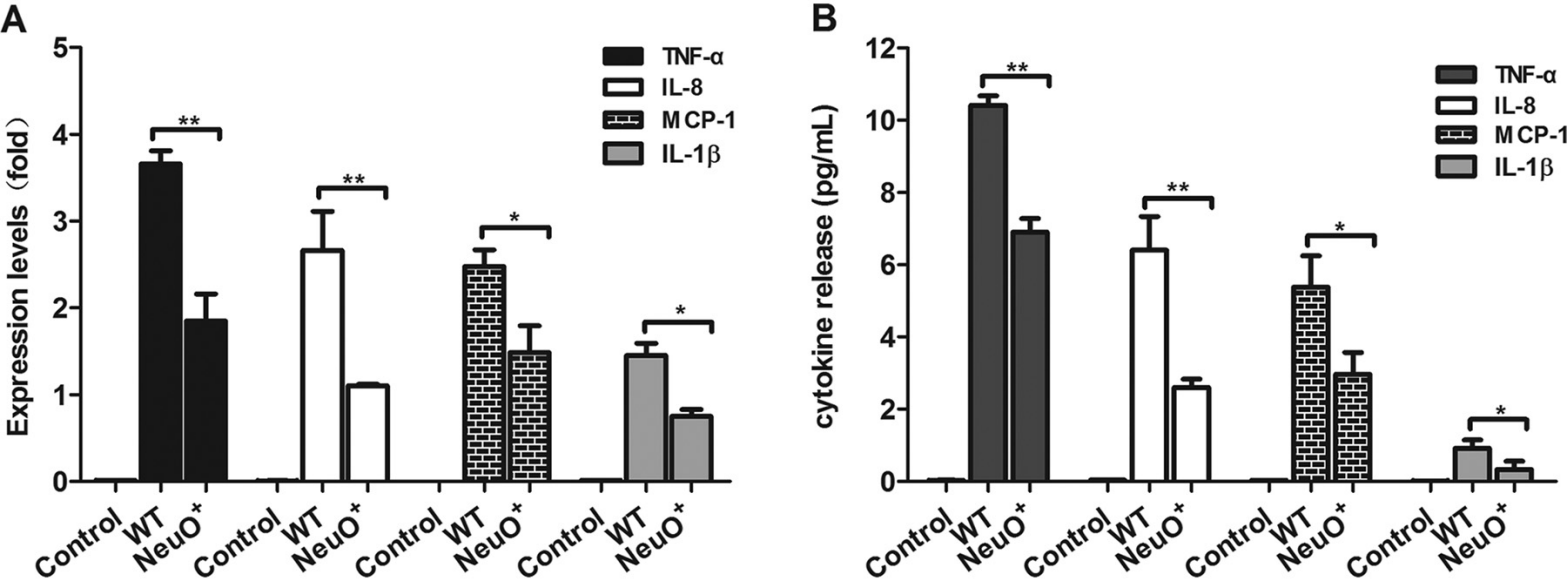
Production of cytokines in human macrophage-like cells. (A) Expression of cytokine genes in THP-M cells stimulated for 24 h with 100 ng/mL capsular polysialic acids purified from the WT or NeuO^+^ strains, respectively. The control represented the expression of genes in the cells without PSA stimulation. The mRNA levels of cytokine genes (i.e., TNF-α, IL-8, MCP-1, and IL-1β) were assessed by RT-qPCR and normalized to *β-*actin mRNA. Data are representative of three independent experiments in triplicate. Values are shown as mean ± SD. (B) Secretion of cytokines in culture supernatants was measured by ELISA. The control represented the cytokines in the cell culture without PSA stimulation. Data were representative of three independent experiments in triplicate, and were analyzed by a two-tailed unpaired Student's *t* test. *, *P* < 0.01; **, *P* < 0.001.

Prior studies showed that Siglecs regulated immunological and inflammatory activities of immune cells (17, 18, 24). To determine the influence of *O*-acetylation of PSA on the immune response, THP-M cells were stimulated by purified PSAs. The mRNA of the genes for cytokines were measured by RT-qPCR. The results showed that tumor necrosis factor α (TNF-α), interleukin-8 (IL-8), monocyte chemotactic protein-1 (MCP-1), and interleukin-1β (IL-1β) were expressed after stimulation by both PSAs (Fig. 7A). The expression levels were highest for TNF-α, medium for IL-8 and MCP-1, and lowest for IL-1β. Remarkably, mRNA production of each cytokine was decreased 2-fold after the stimulation of THP-M cells by the highly *O*-acetylated PSA. Further confirmation was achieved by measuring cytokine levels in supernatants using quantitative enzyme-linked immunosorbent assay (ELISA). The results showed that TNF-α, IL-8, MCP-1, and IL-1β were secreted from THP-M cells at different levels (Fig. 7B). In agreement with mRNA expression, secretions of cytokines were significantly reduced after the administration of highly *O*-acetylated PSA, especially in the case of IL-8. Clearly, *O*-acetylation of K1 capsular polysialic acid suppressed the expression and secretion of proinflammatory cytokines in THP-M cells, thereby weakening the elimination of E. coli K1.

**FIG 8:**
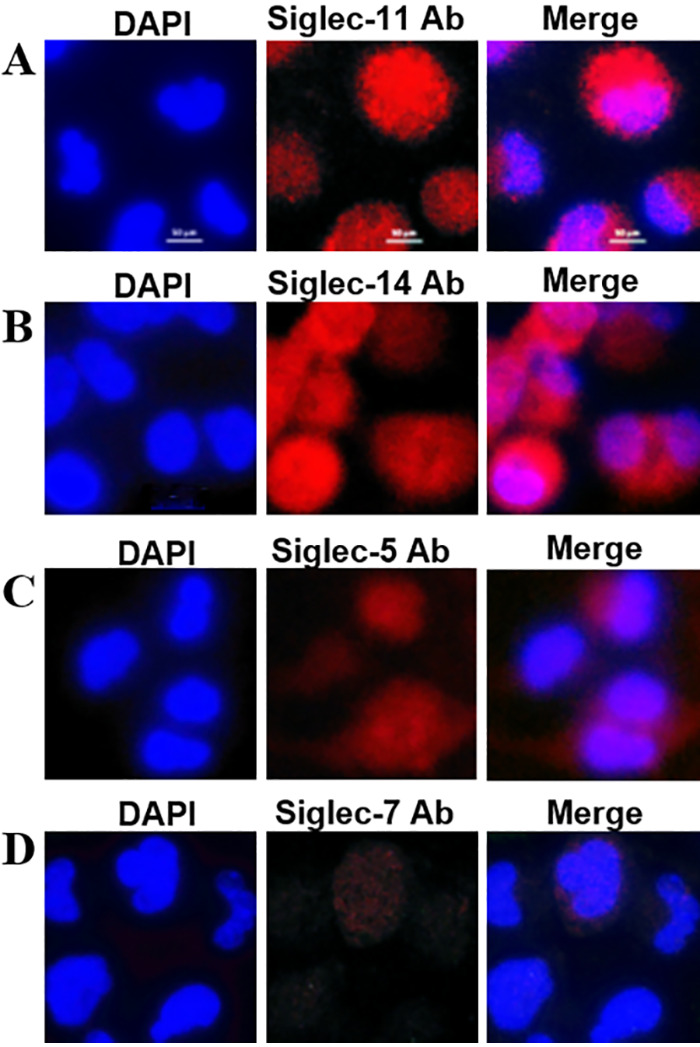
Expression of Siglecs on human macrophage-like cells. THP-1 monocytes were stimulated by PMA for 48 h and differentiated into human macrophage-like cells. These cells were incubated with monoclonal antibodies against human Siglec-11 (A), Siglec-14 (B), Siglec-5 (C), and Siglec-7 (D), and then immunostained with Alexa Fluor 594-conjugated secondary antibodies. Cell nuclei were stained with DAPI. Siglecs expressed on cells were revealed by overlay of images obtained from red and blue channels under a fluorescence microscope. Magnification, ×100; scale bars, 10 μm.

The binding of E. coli K1 to THP-M cells was significantly blocked by the monoclonal antibodies against Siglec-11 and Siglec-14 expressed on the cells. We first detected the Siglecs expressed on THP-M cells using immunocytochemistry immunofluorescence staining. Cells were incubated with the monoclonal antibodies against Siglecs, followed by staining with fluorescence-conjugated second antibody. As expected, Siglec-11, which was naturally expressed on various tissue macrophages, was strongly detected on THP-M cells (Fig. 8A). Surprisingly, the paired Siglec-5/14, which was naturally expressed on neutrophils and monocytes, was also detected on THP-M cells. Obviously, the expression of Siglec-14 was much stronger than that of Siglec-5 (Fig. 8B and C). In addition, Siglec-7, which was naturally expressed on natural killer cells and monocytes, was much less expressed on THP-M cells (Fig. 8D). Therefore, the inhibitory Siglec-11 and the activating Siglec-14 were the main Siglec receptors expressed on THP-M cells.

**FIG 9:**
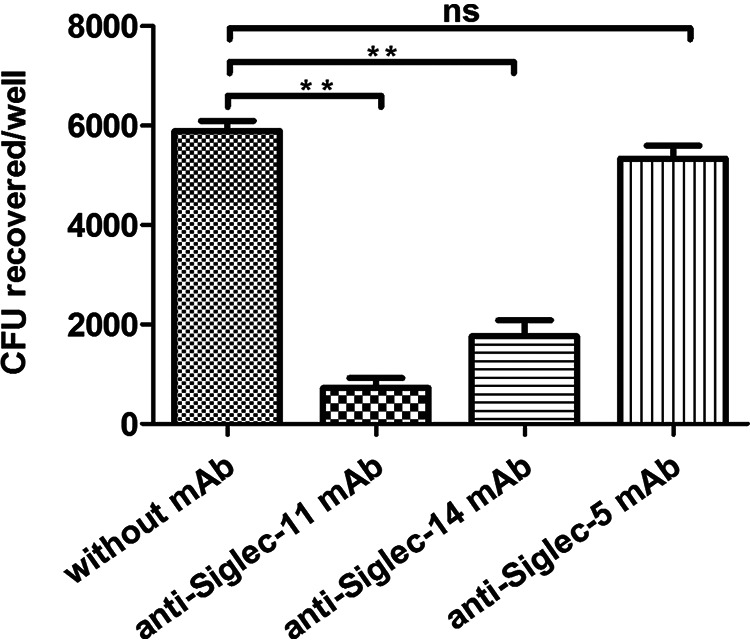
Inhibition of E. coli adherence to human macrophage-like cells by anti-Siglec antibodies. Confluent monolayers of THP-M cells were incubated with monoclonal antibodies against human Siglec-11, Siglec-14, Siglec-5, or with PBS, before addition of the WT. The adherence was carried out as described in Materials and Methods. Each value represents the mean of three independent experiments performed in triplicate, and error bars indicate standard deviations. Data were analyzed by a two-tailed unpaired Student's *t* test. ns, *P* > 0.05; **, *P* < 0.005.

To further verify that Siglec-11 and Siglec-14 on THP-M cells were responsible for interaction with PSAs, THP-M cells were incubated with monoclonal antibodies against Siglec-11, Siglec-14, or Siglec-5, followed by infection of these cells with the WT. The results demonstrated that monoclonal antibodies against Siglec-11 or Siglec-14 effectively inhibited the adherence to THP-M cells by E. coli (Fig. 9). Approximately 87% inhibition was achieved with 20 μg of the antibody against Siglec 11, and 66% inhibition was achieved with 20 μg of the antibody against Siglec 14. However, neither the antibody against Siglec 5 nor the phosphate-buffered saline (PBS) buffer control had any blocking effect. In addition, 5 μg of the antibodies against Siglec 11 or Siglec-14 did not affect adherence, suggesting the inhibition by these antibodies was dose-dependent. These results indicated that Siglec-11 and Siglec-14 identified on THP-M cells were responsible for the interaction with PSAs.

### *O*-acetylation of K1 capsule improved bacteremia and lethality in neonatal mice after intraperitoneal injection.

**FIG 10:**
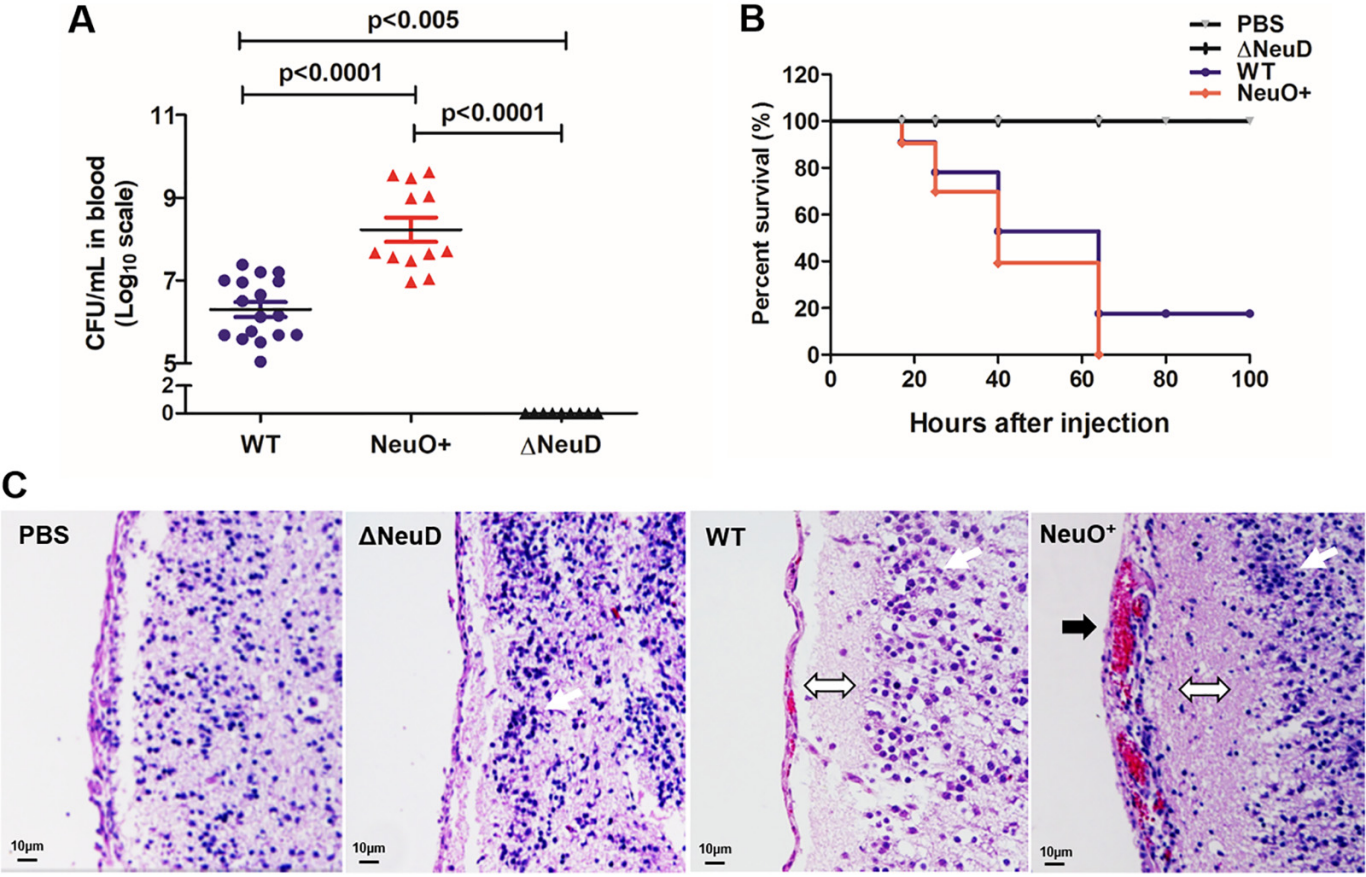
Virulence of different E. coli K1 strains in neonatal mice. (A) Bacterial titers in blood of infected pups. The CD-1 male neonatal mice were injected intraperitoneally with the WT, NeuO^+^, or Δ*neuD* individually. At 17 h postinfection, bacteria in blood taken from tails were plated and counted. Data are presented as mean ± SEM, where *n* = 9 to 11 mice for each group. *P* values are as indicated by an unpaired Student's *t* test with pooled repeats from four independent times. (B) Survival rate of infected pups. Mice were monitored over the course of 5 days for mortality. Statistical comparisons of survival curves were performed by the log rank test, *P* value = 0.019. (C) Brain sections of infected pups. Sectioned brain tissue was stained with H&E for pathological observation under a light microscope. Magnification, ×20; scale bars, 10 μm. White arrow indicates neutrophil filtration, black arrow indicates subarachnoid hemorrhage, and white double arrow indicates enlargement of subarachnoid space.

To determine the effect of *O*-acetylation of K1 capsule *in vivo*, bacteria were injected into neonatal CD-1 mice. After 17 h postinfection, bacterial titers were detected in blood taken from superficial veins in the tails. The data showed that the bacterial CFU in blood infected with the NeuO^+^ was 80 times more than that of blood infected with the WT while the Δ*neuD* was not recovered (Fig. 10A). Moreover, the NeuO^+^ caused more severe infection in the mouse model. After 60 h postinfection, all mice injected with the NeuO^+^ had been killed, 20% of mice injected with the WT survived, and all mice injected with the Δ*neuD* mutant survived (Fig. 10B). The difference in survival between the WT and the NeuO^+^ infection groups is significant (*P* = 0.019) as shown by the log rank test. The brain sections infected by the WT or the NeuO^+^ showed pathological characteristics of meningitis, such as enlargement of the subarachnoid space and inflammatory cell infiltration (Fig. 10C), while the NeuO^+^ caused more severe meningitis symptoms such as subarachnoid hemorrhage. These results revealed that *O*-acetylation of K1 capsule enhanced the level of bacteremia and the rate of lethality in neonatal mice following intraperitoneal bacteria injection.

## DISCUSSION

Meningitis caused by E. coli K1 leads to devastating neurological disability and mortality rates as high as 40% (41–43). As the critical virulence factor, K1 capsular polysialic acid covers bacteria and is frequently modified by *O*-acetyl moieties. However, little is known about the impact of *O*-acetylation of K1 capsule during an infection. In this study, we elucidated the function and underlying mechanism of *O*-acetylation-mediated immune escape of E. coli K1.

The PSA of E. coli K1 is synthesized from CMP-Neu5Ac, an active form of Neu5Ac, and is catalyzed by CMP-Neu5Ac synthetase NeuA. Our previous study shows that NeuA is a bi-functional enzyme possessing CMP-Neu5Ac synthetase and *O*-acetylhydrolase (44). Further study confirms that the biosynthesis pathway of PSA in E. coli is initiated by the *O*-acetylation of free Neu5Ac by *O*-acetyltransferase NeuD, followed by the activation of *O*-acetyl-Neu5Ac by the CMP-Neu5Ac synthetase domain of NeuA, and then de-*O*-acetylation to generate CMP-Neu5Ac by the *O*-acetylhydrolase domain of NeuA, instead of direct activation of free Neu5Ac (45). Thus, the Δ*neuD* mutant loses all K1 capsule (Fig. 1E). Indeed, based on our assay, all free Neu5Ac are *O*-acetylated prior to activation, and most CMP-*O*-acetyl-Neu5Ac are recycled into CMP-Neu5Ac before incorporation into PSA; only a small amount of CMP-*O*-acetyl-Neu5Ac is incorporated into polysaccharide and accounts for a natural 2 to 4% *O*-acetylation, the biological significance of which is not understood. Somehow, there is no doubt that the *O*-acetylation of PSA plays an important role.

The *O*-acetylation of K1 capsule is mainly regulated by the poly-ψ domain of *neuO* carried on E. coli K1-specific prophage CUS-3. Usually, the phase-on strains contain 14 to 39 copies of tandem repeats, and the catalytic efficiency of acetyltransferase increases linearly with increasing numbers of repeats, causing the high frequency of phase variation in E. coli K1 (32, 33). However, it is still unclear what leads to the changes in tandem repeats. The *neuO* gene of the variant contains 39 copies of tandem repeats, suggesting that it makes acetyltransferase at the highest rate of activity. Our studies demonstrated that this prophage-carried *neuO* controlled *O*-acetylation of K1 capsule, which modulated bacterial defense against innate immunity. Similarly, the modification of *O*-antigen of Shigella flexneri by phage-encoded glucosyltransferase promotes bacterial invasion and evasion of innate immunity (46). Therefore, it is likely that inducible enzyme from phage is a “secret weapon” of bacteria that can be activated by certain environmental stimuli or host factors, and that it enables bacteria to evade innate immunity under certain conditions.

Previous studies have shown that E. coli K1 adherence to human brain micro-vessel endothelial cells stimulates the activation of host molecules, resulting in actin polymerization at the adhesion site so that bacteria are internalized into cells by micropinocytosis (39, 47). In order to access the role of *O*-acetylation of K1 capsule in the internalization of bacteria, we examined and compared the entries of the WT, NeuO^+^, and Δ*neuD* strains into cells. Consequently, the internalization of all these strains is inhibited following pretreatment of cells with 1 μg/mL cytochalsin D and 0.5 μg/mL nocodazole, both of which are inhibitors of actin polymerization and cytoskeleton arrangement (see Fig. S5). Moreover, the internalization rates of these strains are nearly identical to their adherence rates, correspondingly (Fig. 2). These results suggest that *O*-acetyl modification of K1 capsule influences bacterial adherence, but has no effect on the bacterial invasion of macrophage-like cells by micropinocytosis.

We present, for the first time, direct evidence that the purified α2,8-linked PSAs interact with human Siglec-5, Siglec-7, Siglec-11, and Siglec-14, but that highly *O*-acetylated PSAs reduce this interaction up to 3 to 4 times (Fig. 6). We also demonstrate that *O*-acetyl moieties are almost equally distributed to the C7 and C9 positions of sialic acids (Fig. 1D). The computer-modeling of Neu5Ac (α2,8) Neu5Ac of ganglioside shows that the 7-*O*-acetyl group is adjacent to the terminal sialic acid, but the 9-*O*-acetyl group is protruding (15). It is known that sialylated molecules recognize a critical arginine residue in the shallow pocket of the outmost V-set domain of Siglec (17). Therefore, it is possible that 9-*O*-acetyl groups hinder the contact of sialic acids to this arginine, and that more *O*-acetyl moieties lead to less avidity of PSA to Siglec.

Each Siglec has a unique specificity for sialyated ligands and shows a characteristic specificity profile (17). On the other hand, Siglecs have an overlapping specificity for such sialic acid-containing glycans. Although 15 human Siglecs have been identified, only Siglec-5, Siglec-7, Siglec-11, and Siglec-14 are able to bind to α2,8-linked polysialic acids with different affinities, suggesting that these Siglecs might share the highest similarity in the V-set domain. In addition, different Siglecs are expressed on different immune cells. For example, Siglec-5 and Siglec-14 are naturally present on monocytes. THP-M cells are differentiated into macrophage-like cells from THP-1 monocytes. We found that Siglec-11 and Siglec-14 are mainly expressed on the surface of THP-M cells (Fig. 8). The result is consistent with the fact that Siglec-11 is naturally present on human macrophages. The expression of Siglec-14 might be due to that the macrophage-like cells are not completely differentiated into macrophages and have the profiles of monocytes.

We further found that the stimulation of THP-M cells by α2,8-linked PSAs induces the production of proinflammatory cytokines, including TNF-α, IL-8, MCP-1, and IL-1β. In contrast, stimulation with highly *O*-acetylated PSAs significantly suppresses the production of these cytokines (Fig. 7). We demonstrated that the adherence of E. coli K1 to THP-M cells can be markedly inhibited by monoclonal antibodies against the two Siglecs, suggesting that Siglec-11 and Siglec-14 are the major receptors on THP-M cells recognized by PSAs (Fig. 9). The ITIM-bearing inhibitory Siglec-11 is involved in anti-inflammatory signal, which leads to the subversion of host immune responses (17, 48, 49). However, the activating Siglec-14 interacts with DAP12, an ITAM-containing adaptor, which triggers proinflammatory signal and enhances the elimination of bacteria (21, 50). It is also shown that expressing Siglec-14 on THP-1 monocytes increases the secretion of TNF-α and IL-8, thus enhancing responsiveness to LPS and Group B Streptococcus (22). We proposed that *O*-acetylation might primarily interfere with the binding of PSA to activating Siglecs on THP-M cells, and negatively regulate the Siglec-mediated kinase signaling cascade, to suppress cytokine secretion and impair the elimination of E. coli K1. It is reasonable to conclude that *O*-acetylation of PSA is an additional subtle modulation to Siglec-mediated host immune response and that it affects infection by E. coli K1.

We demonstrated that *O*-acetylation of PSA increased the virulence of E. coli K1. This result is consistent with clinical observations, which have shown that high-level *O*-acetylated K1 strains are associated with patients with sepsis and bacteremia (7). However, our results disagree with earlier findings which assumed that *O*-acetylated E. coli K1 strains are less virulent due to greater immunogenicity (9, 10). In addition, only de-*O*-acetylated bacteria were recovered from the blood of rat pups fed with E. coli K1 containing 5% O-acetylated PSA (51). The difference suggests that the influence of *O*-acetylation on bacterial virulence depends on the degree of *O*-acetylation as well as the site of infection.

In conclusion, we elucidate that *O*-acetylation of K1 capsule is able to modulate Siglec-mediated adherence and immune response, as well as capsule-mediated ECV trafficking in macrophage-like cells, to enhance the virulence of E. coli K1. It will be of considerable interest in the future to investigate how the degree of *O*-acetylation of K1 capsule is regulated and, in turn, modulates the host immune response to pathogen through Siglecs.

## MATERIALS AND METHODS

### Bacteria strains and plasmids.

The strains and plasmids used in this study are listed in Table 1. E. coli reference strain U9-14 (serotype O2:K1:H4) was obtained from the National Center for Medical Culture Collections in China (code no. 44277). The strain DH5α was purchased from the Dingguo Biotechnology Development Center (China). The plasmids pKD46, pKD3 and pCP20 were purchased from Biovector Science Lab (China). E. coli strains were grown in LB broth. Ampicillin at 100 μg/mL and chloramphenicol at 25 μg/mL were added into media as needed for cultivation of antibiotic-resistant strain.

**TABLE 1:** Plasmids and strains used in this study^*a*^

Plasmid or strain	Description	Source or reference
Plasmid		
pKD3	Template plasmid for RED system, Cm^R^	(52)
pKD46	RED expression plasmid, Amp^r^	(52)
pCP20	Flp expression plasmid, Amp^r^	(52)
		
Strain		
WT	*Escherichia coli* reference strain U9-14, serotype (O2:K1:H4), 3% *O*-acetylation	This study
Δ*neuD*	Mutant of *E. coli* U9-14 in which *neuD* gene was knocked out and lack of K1 capsule	This study
NeuO^+^	Variant of *Escherichia coli* U9-14, K1 capsule with 44% *O*-acetylation	This study

a Cm^R^, chloramphenicol resistance; Amp^r^, antibiotic-resistant bacteria/ampicillin resistance.

### Cell lines.

The murine macrophage-like cell line, RAW 264.7, and human THP-1 monocytes were obtained from the cell bank of the Chinese Academy of Sciences (derived from ATCC). RAW 264.7 cells were maintained in Dulbecco’s modified Eagle’s medium (DMEM, Macgene) supplemented with 10% fetal bovine serum (FBS, Gibco), 100 units/mL penicillin/streptomycin, 4.5 g/L glucose, 1 mM sodium pyruvate, 2 mM l-glutamine, and 25 mM HEPES. The THP-1 cell line was maintained in RPMI 1640 (HyClone) supplemented with 10% FBS, 100 units/mL penicillin/streptomycin, 2.05 mM l-glutamine, and 5 × 10^−5^ M mercaptoethanol (Invitrogen). Both cell lines were cultivated in a humidified atmosphere containing 5% CO_2_ at 37°C. THP-1 monocytes were differentiated into human macrophage-like cells (THP-M) by incubating cells with 100 nM porbol myristate acetate (PMA; Sigma-Aldrich) for 48 h.

### Acquisition of the NeuO^+^ variant.

The contingency locus *neuO* was genetically linked to the endosialidase protein gene carried on prophage CUS-3. To obtain the nucleotide sequences of *neuO* genes, the primers NeuO-1 and NeuO-2 (see Table S1) were designed using Vector NTI software v. 11.5 (Invitrogen) based on the gene *sialK1* and *int* of E. coli strain S88 (GenBank accession no. CU928161.2). The WT was grown in LB broth at 37°C overnight and then transferred into fresh LB broth with 1:100 dilution for propagation. After the passage of the WT four times, the suspension was serially diluted and plated on LB agar plates. The single colonies were picked up and the released DNA were used as the template for PCR amplification using the primers NeuO-1 and NeuO-2. To find the variant isolates, amplicons were sequenced and the nucleotide sequence of *neuO* was compared with that of the WT by Vector NTI v. 11.5 (Invitrogen). The NeuO^+^ variant, in which *neuO* gene contained 39 copies of tandem repeats, was found.

### Acquisition of the Δ*neuD* mutant.

To obtain a mutant which lacked capsule, the *neuD* gene was deleted using the λ Red recombination system of phage lambda according to the previous description (52). Briefly, DNA with a Flp recombination target (FRT)-flanked chloramphenicol resistant (*cat*) gene was generated from pKD3 by PCR amplification with KOD Hot Start DNA polymerase (Millipore), and overlapped with the upstream and downstream fragments of *neuD* gene generated from genome DNA of E. coli strain U9-14 (WT) using the primers from KO-1 to KO-6 (Table S1). The overlapped PCR products were transformed into the WT carrying pKD46, and the chloramphenicol-resistant (Cm^R^) transformants were selected after induction of RED genes. PCR with primers flanking for *cat* gene was carried out and the replacement was confirmed via DNA sequencing. To eliminate the *cat* gene, Cm^R^ mutants were transformed with the Flp recombinase-expressing vector pCP20; the mutant was selected at 30°C, after which colonies were purified once nonselectively at 43°C and then tested for the loss of all antibiotic resistances. The final *neuD*-deleted mutant (Δ*neuD*) was confirmed by PCR and DNA sequencing.

### Quantification of polysialic acids and *O*-acetylation.

Sialic acids and *O*-acetyl groups of K1 capsule were determined by DMB-HPLC analysis as previously described (53, 54). Briefly, 1-mL log phase cultures of the WT and the NeuO^+^ were pelleted and washed four times with PBS. After incubation at 37°C for 1 h with intermittent shaking, capsules were released into the supernatant and collected by centrifugation. The capsules were then completely hydrolyzed in 100* *μL 2 N acetic acid at 80°C for 3 h and lyophilized. The sialic acid residues and the Neu5Ac standards from a Sigma DMB sialic acid labeling kit (ProZyme) were dissolved into 10* *μL water and derivatized with 7 mM DMB (1,2-diamino-4,5-methylenedioxybenzene) reagent following the manufacturer’s protocol. Derivatized sialic acids were analyzed with a reverse-phase GlycoSep R HPLC C_18_ column (ProZyme) in a Shimadzu LC-20AT prominence HPLC. Derived sialic acids were eluted with 40 min isocratic elution in 9% acetonitrile and 7% methanol in water at a flow rate of 0.9 mL/min. The sialic acids were detected by fluorescence detection using a Shimadzu RF-10 AXL Fluorescence Detector (extinction 373 nm and emission 448 nm). The derivatized Neu5Ac standards were used for peak assignment. Integration of HPLC peaks was performed by Shimadzu LC Solution software package to allow the calculation of total sialic acids and *O*-acetylation. The intracellular sialic acids were obtained as previously described, with some modifications (55). The bacterial cells were sonicated followed by centrifugation to remove debris. The sialic acids were collected by precipitation with four volumes of ethanol at −20°C overnight, and centrifuged at 16,000 × *g* at 4°C for 40 min. The precipitates were hydrolyzed, derivatized, and analyzed as described above. All analyses were performed in triplicate and expressed as mean ± standard deviation. Some samples were treated with 0.1 M NaOH at 37°C for 30 min to hydrolyze *O*-acetyl esters, followed by neutralization with 0.1 M HCl.

### Adherence and invasion assay.

The assay was performed as previously described, with some modifications (39). RAW 264.7 cells or THP-M cells were plated on 24-well plates (Corning Incorporated, Costar) in DMEM or RPMI 1640 complete medium, 10^5^ cells/well. Bacteria were added to the cell culture at a multiplicity of infection (MOI) of 1 for RAW 264.7 and a MOI of 10 for THP-M, and incubated at 37°C for 1 h. The monolayers were washed three times with PBS and incubated in medium containing gentamicin (200 μg/mL) for 1 h to kill the extracellular bacteria. The monolayers were washed and lysed with 0.5% Triton X-100. The released intracellular bacteria were enumerated by plating on blood agar plates. To determine the number of total adherent bacteria on cells, the gentamicin step was omitted in the experiments described above. Each set was run in triplicate. For the inhibition assay, cytochalasin D (0.1 and 1.0 μg/mL), nocodazole (0.5 and 5.0 μg/mL), or dimethyl sulfoxide was incubated with cells for 30 min before the monolayers were infected with bacteria. The chemicals were present throughout the experiment.

### Tracking of E. coli K1-containing vacuoles.

RAW 264.7 cells or THP-M cells, respectively, were incubated with FITC-labeled bacteria at a MOI of 100 on Lab-Tek II chamber slides. The trafficking assay was performed as described previously (38). Briefly, at 30 min incubation, the cells were washed, fixed, permeabilized, and probed with the primary antibody against early endosomal marker EEA1. At 60 min incubation, cells were probed with the antibody against the late endosomal marker, Rab7, or the pre-lysosomal marker, Lamp-1. At 90 min incubation, cells were probed with the antibody against the lysozyme marker, cathepsin D. These mouse monoclonal antibodies (Santa Cruz Biotechnology) were used in dilution 1:300 as recommended by the manufacturer. Alexa Fluor 594-conjugated goat anti-mouse IgG antibody (1:250; Abcam) was used to stain the marker proteins for 1 h. After washing with PBS, the slides were mounted in Vecta shield mounting medium with 4′,6-diamidino-2-phenylindole (DAPI; Vector), and viewed with a Fluorescent Microscope Imager A2 with a 100× (1.3 oil) objective (Carl Zeiss AG). The images were obtained and analyzed by Zen 2011 Lite software (Zeiss). Each set was run in triplicate.

### Epitope-specific flow cytometry assay.

This analysis was performed as previously described, with the following modifications (56, 57). For this analysis, 4 × 10^6^ RAW264.7 or THP-M cells were incubated with either the FITC-labeled WT strain or the NeuO^+^ strain, at a MOI of 200, in the dark, at 37°C for 90 min. The cells were extensively washed with ice-cold PBS to remove bacteria, scraped with a cell lifter, and then gently homogenized in 2 mL homogenization buffer. The homogenates were centrifuged three times at 100 × *g* for 5 min to obtain a PNS. For immunostaining of E. coli K1-containing vacuoles, 200 μL of PNS was mixed with 50 μL of normal goat serum and incubated for 1 h on ice with 1 μL of cathepsin D mouse monoclonal IgG (Santa Cruz Biotechnology), a marker protein on lysosome. The samples were incubated with Alexa 594-conjugated goat anti-mouse IgG (1:250 dilution; Abcam) for a further 30 min, and then diluted with 250 μL of PBS. The data were acquired on a BD LSRFortessa flow cytometer and analyzed using FACSDIVA software (Biosciences). The PNS prepared from the cells infected with E. coli was used to set the gate for determining self-fluorescence, and acted as a negative control without red and green fluorescence. The PNS prepared from the cells infected with FITC-labeled bacteria contained a fluorescent population and was used to set the gating threshold of green line. The PNS prepared from the cells infected with E. coli was incubated with monoclonal antibodies followed by A549-conjugated goat anti-mouse IgG, and was used to set the gating threshold of red line. To detect the fusion of ECVs with lysosomes, PNS prepared from cells infected with FITC-labeled bacteria was immunostained with monoclonal antibodies against cathepsin D, and revealed with A549-conjugated secondary antibodies before the flow cytometry analysis. PNS containing both green and red fluorescent population of particles indicated the fusion of ECVs with lysosomes.

### Determination of the expression and secretion of cytokines.

THP-M (10^6^ cells) were incubated with 100 ng/mL polysialic acids, purified from either the WT or the NeuO^+^, for 24 h. The cells were collected for RNA extraction and the supernatants were collected for performing an ELISA. The cell culture without PSA stimulation acted as a control. Total RNA was extracted by a RNAprep Pure Cell/Bacteria kit (Tiangen Biotech, China), and cDNA was synthesized by a FastQuant RT kit with gDNase (Tiangen Biotech, China) according to the manufacturer’s guidelines. Using the primers listed in Table S1, quantitative RT-PCR (RT-qPCR) was performed with a KAPA Syber Fast qPCR kit (KAPA Biosystems) following the product protocol, using the C1000 Touch Cycler instrument (Bio-Rad). Measurement was performed in triplicate and the results were detected by CFX96 Optimal Reaction Module. The expression levels of genes for cytokines (i.e., TNF-α, IL-8, IL-1β, and MCP-1) relative to β-actin were analyzed by CFX96 Manager software (Bio-Rad) using the 2^−ΔΔCt^ method (58). Cytokine proteins were measured by an ELISA Ready-Set-Go Kit, including a human TNF-α uncoated ELISA set (Invitrogen). Each assay was performed according to the manufacturer’s instructions and the plate was read at OD_450_ nm using a SpectraMax Paradigm Multi-Mode Detection Platform (Molecular Devices). The amount of cytokine was calculated based on the standard curve, which was generated from the recombinant protein in this kit.

### Binding of rhSiglec-Fc chimeras with bacteria.

The assay was performed as previously described (59). Immulon 4HB ultrahigh binding microtiter plates (Thermo Scientific) were coated with 0.5 mg/mL protein A in coating buffer at 4°C overnight. Wells were washed three times and blocked with assay buffer. Human CD33 rSiglec-Fc chimeras (R & D Systems) were added to wells at 0.5 mg/mL and adhered at 4°C overnight. Wells were washed three times with assay buffer. 10^7^ FITC-labeled bacteria suspended in 50* *μL assay buffer were added into the well and incubated at 37°C for 10 min. After washing, the fluorescent intensity (excitation 488 nm, emission 530 nm) was measured using a SpectraMax Paradigm Multi-Mode Detection Platform (Molecular Devices).

### Isolation of PSAs and examination of PSAs.

The isolation and purification of capsular polysaccharides were performed as previously described, with some modifications (60). E. coli culture in LB medium was gently stirred in a 37°C water bath to release capsules. The supernatants were collected and concentrated with a 30 kDa PLTK ultrafiltration membrane (Millipore). The retained high-molecular-weight fraction was harvested. The nucleotide acids were precipitated with 25% cold ethanol for 10 min and then removed. Capsular polysaccharides were precipitated by 75% cold ethanol at 4°C overnight. The precipitations were collected and dissolved into 10% sodium acetate. Proteins were removed by treatment with 2-fold cold phenol. The crude PSAs were dialyzed against 0.1% CaCl_2_ and water followed by 10 mM Tris-HCl buffer (pH 8.0) containing 10 mM NaCl, and then applied to a DEAE Sephacel (GE Healthcare) anion exchange column. The column was eluted with a linear gradient of NaCl (10 to 100 mM) in Tris-HCl buffer. Fractions were collected and monitored by phenol sulfuric acid reaction. The pure PSA in the fractions was pooled and dialyzed against water, followed by lyophilization.

PSA were solubilized with ultrapure water and analyzed by PAGE comprising 5% spacer and 7% separation gel using the sodium borate-sodium hydrate running buffer (pH 9.4). The PageRuler Prestained Protein Ladder (Thermo Scientific) was used as a marker. The gel was then stained with a Glycoprotein Staining Kit (Thermo Scientific) following the manufacturer’s protocol. The molecular weight of PSA was estimated using HPGPC with Ultrahydrogel 120, 250, and 1000 connected columns (Waters) and a refractive index detector. The PSAs were eluted with mobile phase containing 0.1 M NaNO_3_ and 0.05% NaN_3_ at a flow rate of 0.5 mL/min. To obtain a standard curve to estimate molecular weight of PSA, the column was calibrated by standard dextrans (100 Da, 500 Da, 5,200 Da, 48.6 kDa, and 668 kDa), and retention times were plotted against molecular weights on a logarithmic scale. All samples were prepared as 5 mg/mL solutions, and 20* *μL of solution was analyzed in each run.

We also identified the *O*-acetyl groups of the two PSAs by ^1^H NMR analysis. NMR spectra were recorded on a Bruker Avance III 500 at 25°C. The purified PSAs (5 mg) were exchanged in D_2_O (99.8% atom D) by three cycles of lyophilization from this liquid. Dried samples were then dissolved in 0.5 mL of D_2_O for NMR. To quantify the contents of *O*-acetyl groups, 1 mg compound caffeine was added into each of the samples as an internal standard. The spectrum was acquired at 500 MHz for ^1^H. All protone chemical shifts were reported relative to tetramethyl silane.

To examine whether the purified PSAs were free of lipid-A, we performed ^31^P NMR for these PSAs. It is well known that LPS contains lipid-A, which has glycosidic diphosphate moiety. Because PSAs only contain sialic acids, LPS was able to serve as a positive control in this analysis. ^31^P NMR spectra were recorded on a Bruker Avance III 500 at 25°C. The PSAs (20 mg) and LPS (20 mg; Solarbio Life Sciences) were dissolved in 0.5 mL deionized water and sonicated until a uniform solution was obtained for NMR analysis. The spectrum was acquired at 202.4 MHz over 32 accumulations. The chemical shifts were measured relative to the external 85% phosphoric acid.

### Quartz crystal microbalance assay.

The interaction between purified PSA and rSiglec-Fc chimeras was measured using an Attana Cell A200 QCM Biosensor (Attana AB) as previously described (61). First, the rSiglec-Fc chimeras were immobilized on the 2D carboxyl dextran sensor surface. The chip surface was activated twice with a 1:1 mixture of 0.2 M EDC and 0.05 M sulfo-NHS (*N*-hydroxysuccinimide) for 10 min. The rSiglec-Fc was dissolved into 10 mM acetic acid buffer (pH 4.5) at a concentration of 50 g/mL, and injected three times over the activated chip surface. To deactivate any remaining NHS esters, two injections of 1 M ethanolamine (pH 8.5) were performed for 10 min each. The surface sensor was then inserted into the QCM Biosensor and stabilized under a continuous flow (20* *μL/min) of PBS running buffer. The measurements were initiated when the resonant frequency was stable. The purified PSA (10 μM) was injected over the surface of the chips, and binding between PSA and rSiglec-Fc chimeras was monitored for association for 85 s and dissociation for 300 s. The resonant frequency of the quartz crystal and the frequency shift (Δ*f*) associated with association or dissociation were recorded with Attester software in real time. Following each association and dissociation cycle, the cell chips were regenerated between measurements by 1 to 2 injections of 10 mM glycine (pH 2.0) or 300 mM corresponding PSA to dissociate the bound proteins, and were immediately re-equilibrated with running buffer. The data were then analyzed by Clamp XP software (Attana AB).

### Siglecs expressed on human macrophage-like cells.

THP-1 monocytes (10^5^/well) were differentiated into macrophage-like cells with 100 nM PMA for 48 h in a Lab-Tek II chamber slide (Thermo Fisher). The cells were fixed with 4% paraformaldehyde and blocked in the buffer containing 1% BSA, 10% normal goat serum, and 0.3 M glycine in PBS for 1 h at room temperature. The cells were stained with 1 μg/mL monoclonal antibodies against human Siglec-11, Siglec-14, Siglec-5, and Siglec-7 (R & D Systems), respectively, overnight at 4°C, and were then probed with 2 μg/mL Alexa Fluor 594 goat-anti-mouse secondary antibody (Abcam) at room temperature for 1 h. After washing with PBS, the slides were mounted in Vecta shield mounting medium with DAPI (Vector), and viewed with a Fluorescent Microscope Imager A2 with a 100× (1.3 oil) objective. The images were obtained by Zen 2011 Lite software. Each set was performed in triplicate.

### Inhibition assay.

THP-1 cells (10^5^/well) were stimulated into macrophage-like cells as described above. The THP-M cells were washed and then incubated with 20 μg monoclonal antibodies against Siglec-11, Siglec-14, and Siglec-5 (R & D Systems) for 3 h at 4°C, respectively. E. coli K1 in log phase (10^7^/well) was added into well and incubated for 1 h at 37°C. The monolayers were washed three times with PBS and treated with 0.5% Triton X-100. The released bacteria were plated on blood agar plates and enumerated. Each set was run in triplicate.

### Mouse infection studies.

The animal studies were approved by the Animal Care and Use Committee of the Institute of Microbiology and followed National Institutes of Health guidelines for the performance of animal experiments. The neonatal CD-1 mouse pups (aged 2 to 3 days, weight 2.5 to 3.7 g; Beijing Vital River Laboratory Animal Technologies Co. Ltd., China) were randomly divided into four groups. In each group, litters of 9 to 11 pups were kept in a cage with the mother mouse. Mice were housed under temperatures of 22 ± 2°C with 50% ± 10% humidity. Pups were infected with 10^5^ CFU of mid-exponential-phase WT, Δ*neuD*, and NeuO^+^ in 25* *μL of saline via intraperitoneal injection. Control mice received saline through the same route. All litter members were infected in an identical fashion and at the same time. Blood was collected from the tail at 17 h postinfection and put into PBS with heparin. Bacterial titers were detected by serial dilution of blood samples, plating on blood agar plates, and counting colonies. The mice were then at 24 h postinfection and their brains were fixed in 4% formaldehyde, dehydrated, and embedded in paraffin as routinely processed. The 4-μm sections were cut on a Leica Microtome and stained with hematoxylin and eosin, and pictures were taken with a Nikon CI-S microscope at ×20 magnification. Animal survival was monitored for 100 h postinfection. The experiment was performed in pooled repeats four independent times. The statistical significance of bacteria titers in blood was evaluated by an unpaired two-tailed Student's *t* test. Statistical comparisons of survival curves were performed by the log rank test.

### Statistical analysis.

In animal experiments, mice were randomly selected and grouped without using specific blinding procedures or exclusion conditions. Numbers of mice in each group and statistical details are indicated in the figure legends. The cell experiments were performed with triplicate samples and in three independent experiments. QCM experiments were performed in three independent experiments. Quantitative data were plotted and analyzed by GraphPad Prism 6 (GraphPad Software). Data are shown as the mean ± standard deviation (SD) or mean ± standard error of the mean (SEM). Differences were analyzed using a two-tailed unpaired Student's *t* test, and a *P* value of <0.05 was considered significant.
