# Supplementary material for: O-Acetylation of Capsular Polysialic Acid Enables Escherichia coli K1 Escaping from Siglec-Mediated Innate Immunity and Lysosomal Degradation of E. coli-Containing Vacuoles in Macrophage-Like Cells
Source: Microbiol Spectr. 2021 Dec 8;9(3):e00399-21. doi: 10.1128/spectrum.00399-21 (PMC8653822; doi:10.1128/spectrum.00399-21)
Supplement: SUPPLEMENTAL FILE 1 — Supplemental material. Download SPECTRUM00399-21_Supp_1_seq16.pdf, PDF file, 0.7 MB [file spectrum00399-21_supp_1_seq16.pdf]

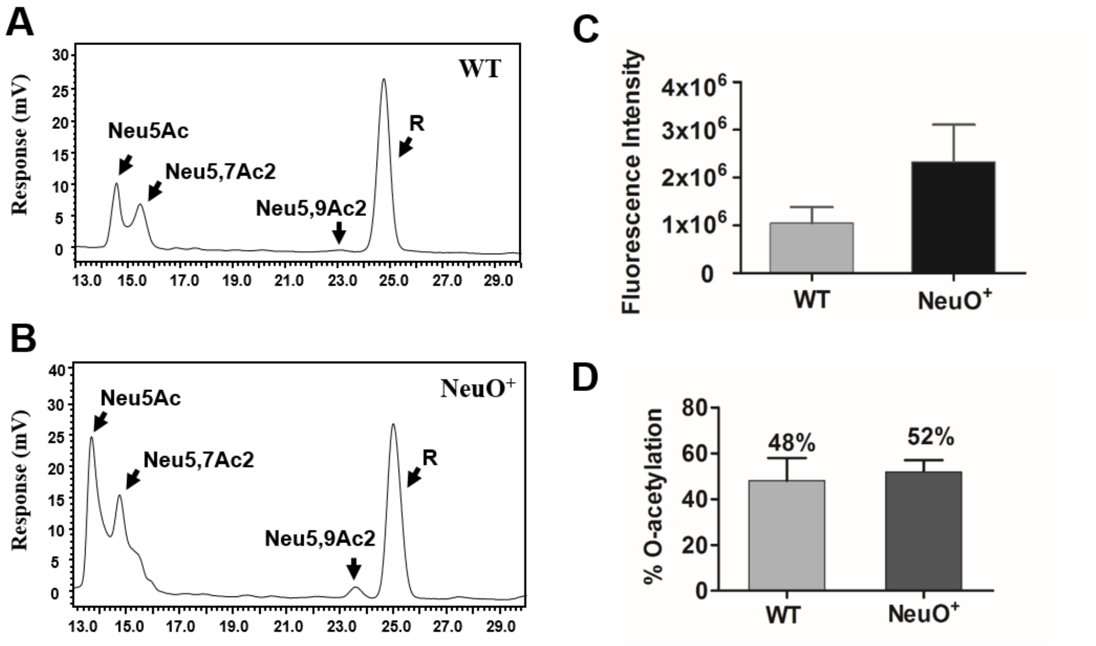

FIG S1 DMB HPLC analysis of intracellular sialic acids from the WT (A) and the NeuO^{+} (B). The peaks are assigned referring to the corresponding standards. (C) Total amounts of intracellular sialic acids of the WT and NeuO^{+}. (D) The level of *O*-acetylation of intracellular sialic acids from the WT and the NeuO^{+}. Data are shown as mean ± SD. Error bars indicates median for values from three or four separate experiments.

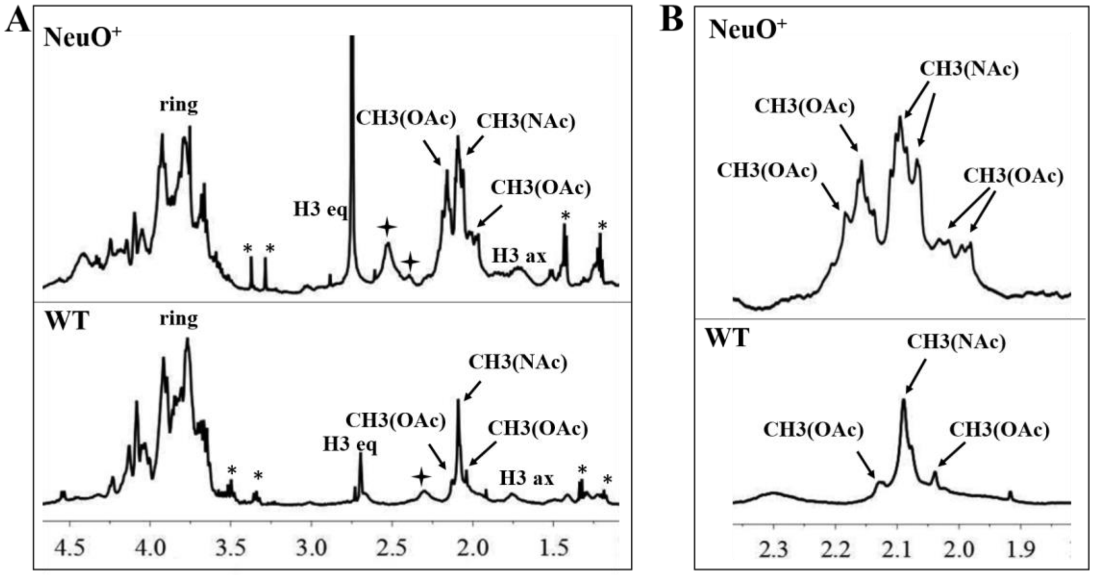

FIG S2. Comparison of one-dimensional ^{1}H NMR spectra of the purified polysialic acids isolated from the WT and $NeuO^{+}$ strains. (A) Expansion of NMR spectra (δ=4.50–1.50 ppm) with assignments of the major resolved signals is indicated, including the methyl protons from carbon 4 to carbon 9 on Neu5Ac ring (δ=4.30–3.50 ppm) and the methyl protons of acetyl groups (δ=2.00-2.15 ppm) and H-3 of Neu5Ac (1.80 ppm and 2.75ppm). Signals marked with asterisks do not originate from carbohydrate material. Signals marked with stars are uncertain materials. (B) The acetyl regions of the two PSAs are amplified. The peaks between 2.07 ppm and 2.09 ppm were assigned *N*-acetyl signals. The peaks between 2.13 ppm and 2.20 ppm, and the peaks between 1.98 ppm and 2.03 ppm were assigned *O*-acetyl signals, respectively. The content of O-acetyl groups in the $NeuO^{+}$ PSA is about ten times that of the WT PSA by comparing with the internal standard compound. Spectra were recorded at 500 MHz at 25°C. All protone chemical shifts (ppm) were reported relative to TMS.

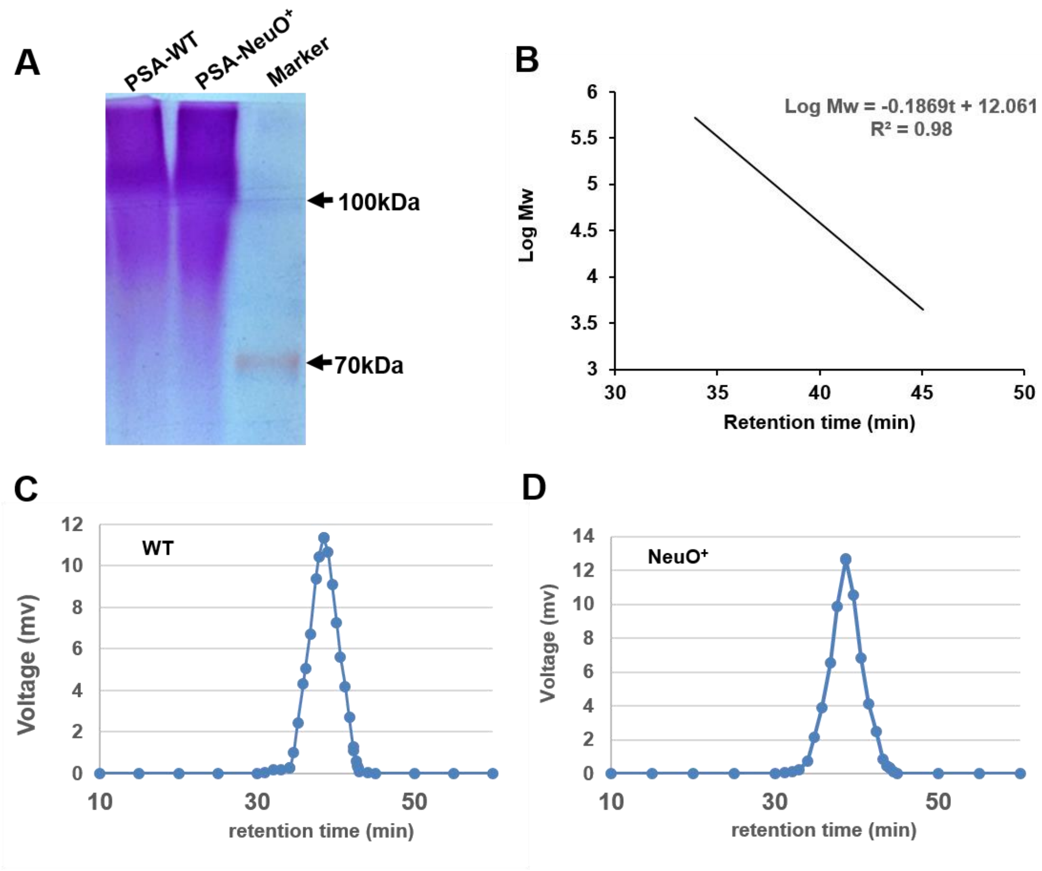

FIG S3 Measurement of molecular weight of PSAs using HPGPC. (A) The PSA was isolated and purified from strains WT and $NeuO^+$, and separated by polyacrylamide gel comprising 5% spacer gel and 7% separation gel, and visualized by glycoprotein staining kit. The PageRuler pre-stained protein ladder (180 kDa) was used as molecular marker. (B) Molecular weight of PSAs were estimated by high-performance gel permeation chromatography (HPGPC) with Waters Ultrahydrogel columns. The standard curve was obtained by calibrating the dextrans and plotted against the molecular weights on a logarithmic scale. (C) PSA of the WT and (D) PSA of the $NeuO^+$ were analyzed by HPGPC chromatograms. The retention times (t) were applied to the standard formula to calculate molecular weight of PSA.

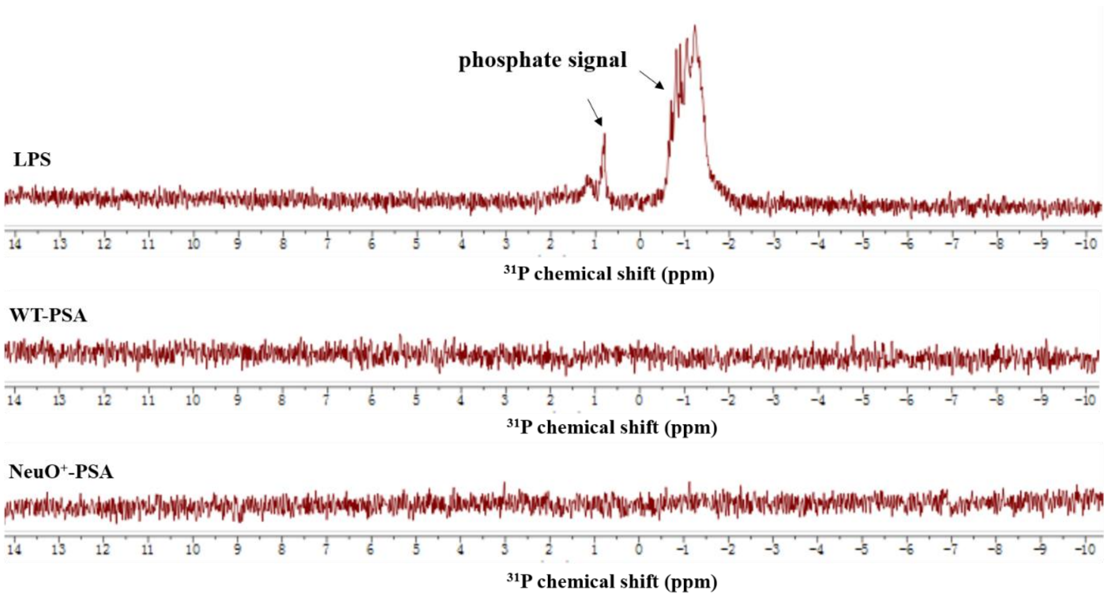

FIG S4 ^{31}P NMR spectra of LPS (A) and polysialic acids (PSAs) from the WT strain (B) and the NeuO$^+$ strain (C). The ^{31}P signals are present in the spectrum of LPS but not in the spectra of PSAs, indicating that the purified PSAs are free of lipid-A. The ^{31}P spectrum was acquired at 80.9 MHz in 35 accumulations. The chemical shifts (ppm) were measured retative to external 85% phosphoric acid.

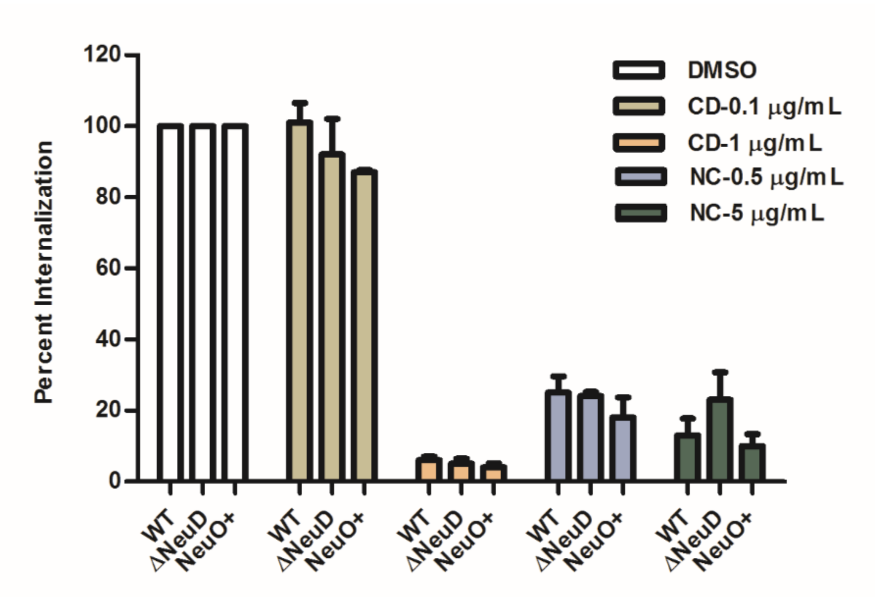

FIG S5 Effects of cytochalasin D (CD) and nocodazole (NC) on internalization of *E. coli* into macrophages. RAW264.7 was treated with inhibitors cytochalasin D and nocodazole in different concentrations prior to the assay. The levels of internalization of the WT, Δ*neuD* and NeuO$^+$ strains were calculated and expressed as relative values. The level of internalization into DMSO-treated cells was defined as 100%. The experiments were carried out at three seperate times in triplicate, and data are shown as mean ± SD.

TABLE S1. The primers used in this study were listed in the table. The names of primers, the sequences of forward primers (F) and reverse primers (R), and the locations were listed. The purpose of these primers was indicated in the manuscript.

TABLE S1. The primers used in this study

**Primers**	**Forward primer (F)) or Reverse primer (R)**	**Location**
KO-1	**5'**-ACGCGTCGACGAAGCCTATGTTATTCT (F)**-3'** (F)	Upstream of *neuD* gene
KO-2	**5'**-ATTTAACTGAGACATATCATGAGTAATATATATATCG-**3'** (R)	Upstream of *neuD* gene
KO-3	**5'**-CGATATATATATTACTCATGATATGTCTCAGTTAAA-**3'** (F)	downstream of *neuD* gene
KO-4	**5'**-ACGCGTCGACACATTGCCCTGATTGGTC-**3'** (R)	downstream of *neuD* gene
KO-5	**5'**-ATGAGTAAAAAATTAATAATATTTGGTGCGGGTGGTTTTTCAAAATTGTAGGCTGGAGCTGCTTC-**3'** (F)	FRT-flanked Cm^r gene
KO-6	**5'**-TCATTCATTCCCCCTAATTAATCTTGTTGGAGTCCCAGCAACTACAACATATGAATATCCTCCTTAGT-**3'** (R)	FRT flanked-Cm^R
NeuO-1	**5'**-CGGGAGCATCATTGTTGATGAG-**3'** (F)	*sialK1* gene
NeuO-2	**5'**-CATGGTTACTTCACTACTTCCGCAC-**3'** (R)	*int* gene
β-actin-1	**5'**-AGCGAGCATCCCCCAAAGTT-**3'** (F)	*β-actin* gene
β-actin-2	**5'**-GGGCACGAAGGCTCATCATT-**3'**(R)	*β-actin* gene
TNF-α-1	**5'**-CCTTCCTGATCGTGGCAG-**3'**(F)	*TNF-α* gene
TNF-α-2	**5'**-GCTTGAGGGTTTGCTACAAC-**3'**(R)	*TNF-α* gene
IL-1β-1	**5'**-TCCCCAGCCCTTTTGTTGAG-**3'**(F)	*IL-1β* gene
IL-1β-2	**5'**-GGAGCGAATGACAGAGGGTT-**3'**(R)	*IL-1β* gene
IL-8-F	**5'**-GCCAACACAGAAATTATTGTAAAGCTT-**3'**(F)	*IL-8* gene
IL-8-R	**5'**-AATTCTCAGCCCTCTTCAAAAACTT-**3'**(R)	*IL-8* gene
MCP-1-F	**5'**-CAGCCAGATGCAATCAATGC-**3'**(F)	*MCP-1* gene
MCP-1-R	**5'**-GTGGTCCATGGAATCCTGAA-**3'**(R)	*MCP-1* gene
